# Chirality Regulates Stem Cell Fate and Promotes Corneal Epithelial Regeneration via Manipulating Notch Pathway

**DOI:** 10.1002/advs.202504732

**Published:** 2025-05-30

**Authors:** Shiding Li, Yu Zhao, Junzhao Chen, Beibei Wu, Nianxuan Wu, Hao Sun, Liangbo Chen, Chuanliang Feng, Yao Fu

**Affiliations:** ^1^ Department of Ophthalmology Shanghai Ninth People's Hospital Shanghai Jiao Tong University School of Medicine Shanghai 200011 China; ^2^ State Key Laboratory of Eye Health Shanghai Jiao Tong University Shanghai 200011 China; ^3^ State Key Lab of Metal Matrix Composites School of Materials Science and Engineering Shanghai Jiao Tong University Shanghai 200240 China

**Keywords:** chiral hydrogel, chirality, corneal epithelial regeneration, limbal epithelial stem cells, Notch signaling pathway

## Abstract

The differentiation and stemness maintenance of stem cells are the core topics in cell biology and regenerative medicine, involving cell fate determination, developmental regulation and tissue regeneration. Chirality is an essential factor influencing multiple biological processes, including protein interactions, stem cell development and disease pathogenesis. However, its roles in regulating stem cells fate, especially limbal epithelial stem cells (LESCs), remain elusive. Herein, it is first discovered that right‐handed chiral hydrogel (DH) enhanced LESCs proliferation, migration, and differentiation into corneal epithelial cells, while left‐handed chiral hydrogel (LH) can partially preserve LESCs stemness. Further in vivo experiments demonstrated that 3D DH effectively accelerated corneal wound healing process, inhibited both inflammation and vascularization in a partial limbal stem cell deficiency model. Mechanistically, DH activates Notch signaling by increasing its stereo‐affinity to Notch1, facilitating Notch intracellular domain release and HES1 transcription, thereby directing LESCs fate. Collectively, this work highlights the novel role of chirality in LESCs fate determination and confirms DH as a drug‐free, effective approach for corneal epithelial regeneration, offering a new direction for regenerative medicine and tissue engineering.

## Graphical abstract

Chirality governs the limbal epithelial stem cell fate. Right‐handed chirality enhances cell proliferation and differentiation, while left‐handed chirality partially maintains cell stemness. By activating the Notch pathway, 3D right‐handed chiral hydrogel offers a drug‐free and cell‐free strategy for effective corneal regeneration. This work unveils a novel link between chirality and stem cell behavior, opening new avenues in corneal regenerative medicine.

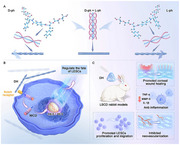

## 1 Introduction

Stem cells are multipotent cells with the ability to self‐replicate and differentiate into a variety of functional cells under certain conditions to promote the regeneration of various tissues and organs.^[^ ^1^, ^2^ ^]^ The differentiation and stemness maintenance of stem cells are two core processes in stem cell biology, which are both opposite and closely related to each other, and jointly determine the fate and behavior of stem cells.^[^ ^3^, ^4^ ^]^ For example, Aguadé‐Gorgorió et al.^[^ ^5^ ^]^ revealed that MYC target 1 maintained the stemness of human haematopoietic stem cells by regulating endocytosis and environmental sensing. Winston et al.^[^ ^6^ ^]^ employed a stepwise human pluripotent stem cell ‐to‐ induced mesenchymal stromal cells  differentiation method via intermediate cell stages of neural crest and cytotrophoblast to generate lineage‐specific mesenchymal stromal cells with varying differentiation efficiencies and gene expression. Understanding the regulatory mechanisms of stem cell fate is essential to reveal the origin and development of organisms, elucidate the etiology of various diseases and promote tissue regeneration.^[^ ^7^, ^8^, ^9^ ^]^

When stem cell function is impaired or their microenvironment is destroyed, stem cells are unable to promote tissue repair, which can lead to some intractable diseases including aplastic anemia and Alzheimer's disease.^[^ ^10^, ^11^ ^]^ Among them, vision‐related diseases have received extra attention due to their significant impact on people's quality of life.^[^ ^12^ ^]^ As the outermost part of the eye, cornea's external location and transparency render it a natural window for vision and to protect its internal structures from trauma and microbes.^[^ ^13^, ^14^, ^15^ ^]^ When the corneal epithelium is injured, quiescent limbal epithelial stem cells (LESCs), a population of stem cells located in a highly regulated niche in the limbus, will activate, proliferate, and differentiate into corneal epithelial cells (CECs) to restore the epithelium.^[^ ^16^, ^17^ ^]^ Damage to LESCs or their niche could lead to limbal stem cell deficiency (LSCD), characterized by corneal opacity, conjunctivization, neovascularization, visual impairment and even blindness.^[^ ^18^ ^]^ Currently, treating LSCD mainly relies on stem cell transplantation, including keratolimbal allograft transplantation, simple limbal epithelial transplantation and cultivated limbal epithelial transplantation.^[^ ^19^, ^20^ ^]^ However, these surgeries encounter the challenges such as insufficient supply of donor tissues and immune rejection, which limit their clinical application.^[^ ^21^, ^22^, ^23^ ^]^ Notably, many LSCD patients retain a small population of residual LESCs, highlighting the urgent need for innovative therapeutic strategies that can effectively promoting the regeneration of LESCs.

At present, numerous studies are focused on developing novel drugs to promote the regeneration of LESCs.^[^ ^24^, ^25^ ^]^ However, instability, dilution and efflux, low bioavailability and potential toxicity greatly restricts their clinical transformation and application. With the further in‐depth understanding of niche microenvironment, increasing evidence has confirmed the interaction and mutual promotion of the microenvironment with LESCs provide the necessary homeostasis for cell growth and determine the fate of LESCs to influence biological processes.^[^ ^26^, ^27^, ^28^, ^29^ ^]^ For instance, the niche stromal cells maintained the stemness of LESCs via membrane receptor‐ligand interaction of SDF‐1/CXCR4.^[^ ^30^ ^]^ In addition, extracellular matrix (ECM) have been proven to influence cell fate and phenotype through YAP/TAZ, β‐catenin signaling and ΔNp63 pathways.^[^ ^31^, ^32^ ^]^

Chirality, as one of the most distinctive features of living organisms and storage forms of biological signals, is widely ubiquitous in the nature and microenvironment, especially in ECM.^[^ ^33^, ^34^, ^35^, ^36^ ^]^ Plentiful basic building blocks of biont, such as proteins, DNA and RNA, are also highly dependent on their chirality, thus involving in a variety of physiological functions and manipulating cell behaviors.^[^ ^37^, ^38^ ^]^ For instance, unique chirality selection in neural cells enables specific manipulation of cell behaviors to promotes sciatic nerve repair by attenuating interaction between matrixes and cytoskeleton proteins, thus activating JNK and p38/MAPK signaling pathways.^[^ ^39^ ^]^ Also, chirality can effectively control mesenchymal stem cell lineage diversification through mechanoresponses, which involve contractile state, focal adhesion kinase/extracellular signal‐regulated kinase 1/2 cascades, and yes‐associated protein/runt‐related transcription factor 2 nuclear translocation.^[^ ^40^ ^]^ More importantly, these biomimetic ECM characteristics are not only closely related to cell microenvironment, but also a favorable structural feature of natural peptides and proteins, which have always been pursued for a long time to act as artificial biomedical materials for tissue engineering and regeneration therapies, involving in musculoskeletal, neurodegenerative, tumor immune response and cardiovascular diseases.^[^ ^39^, ^41^, ^42^, ^43^, ^44^, ^45^, ^46^, ^47^, ^48^, ^49^ ^]^ However, as one of the most significant biophysical factors, the potential role of chiral structure in LESCs and the repair of the ocular surface has not been fully explored. Considering the unique role of chirality in biological processes and microenvironment, investigating the impact of matrix chirality on LESCs via constructing a chiral microenvironment represents a critical scientific endeavor, as well as a novel strategy to manipulate stem cell behaviors and promote tissue regeneration.

Herein, we utilized the enantiomeric L/D‐phenylalanine gelators and their racemic mixture to construct the chiral extracellular microenvironment and investigate their effects on LESCs for the first time. It was discovered that right‐handed chiral hydrogel (DH) enhanced LESCs proliferation, migration and differentiation into CECs, while left‐handed chiral hydrogel (LH) partially preserved LESCs stemness. In addition, by constructing 3D chiral hydrogel, we innovatively found that DH effectively accelerated corneal wound healing process, inhibited inflammation and vascularization in partial LSCD rabbit models through a drug‐free manner. Mechanistically, DH exhibited a stronger stereo‐affinity for Notch1 binding, activating the Notch signaling pathway to promote LESCs proliferation and corneal repair. Collectively, this study offers a compelling foundation for chiral regulation of stem cell fate and novel therapy for corneal epithelial regeneration.

## 2 Results and Discussion

### 2.1 Construction and Characterization of Chiral Hydrogels

**Scheme 1:**
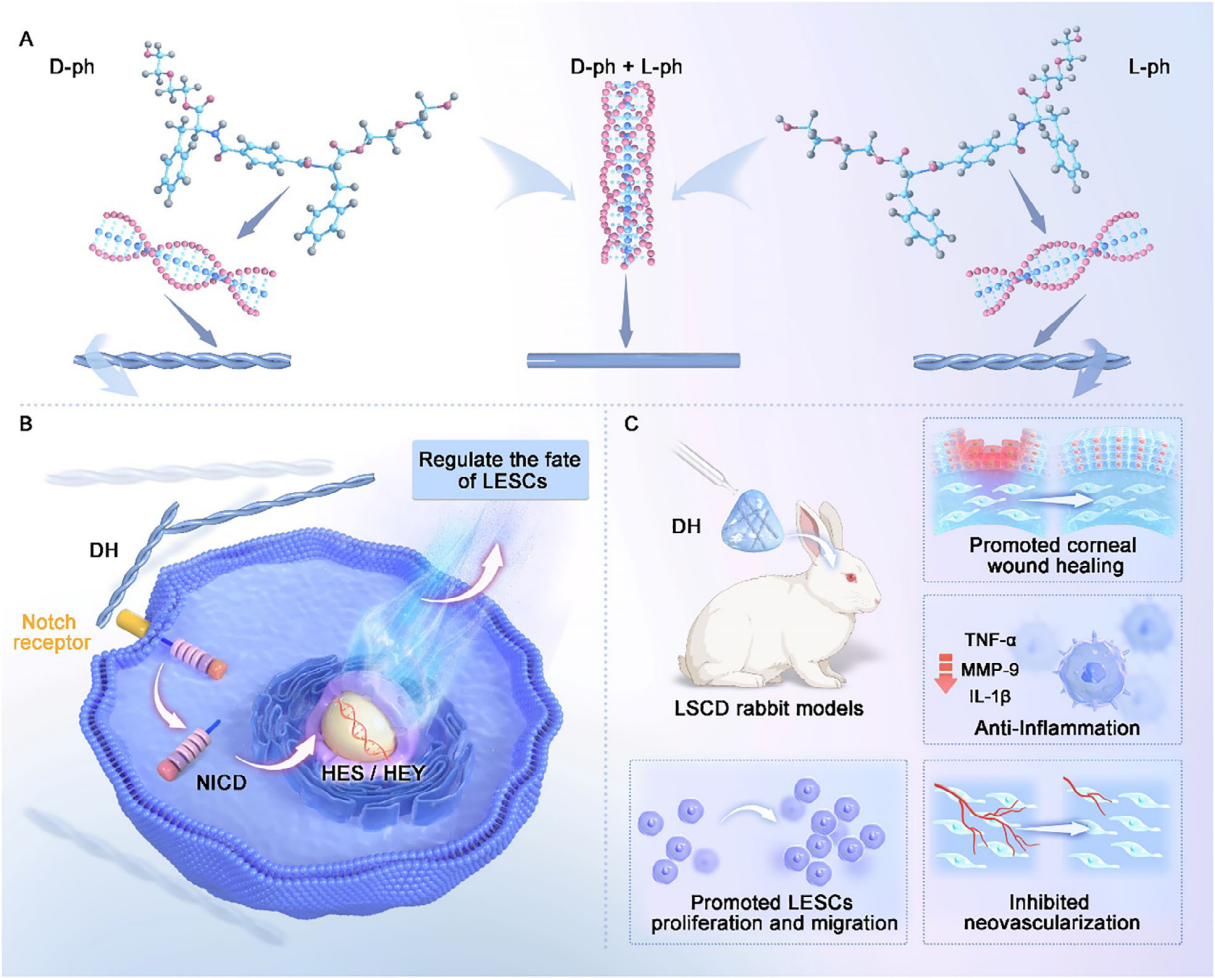
Schematic illustration of chiral hydrogel construction and the regeneration of corneal epithelium by DH. A) Schematic representation illustrating chiral matrix fabrication through self‐assembly. B) Schematic diagram of molecular mechanism in the fate of LESCs manipulated by DH. C) Overview of the application of DH for corneal wound healing and regeneration.

**Figure 1:**
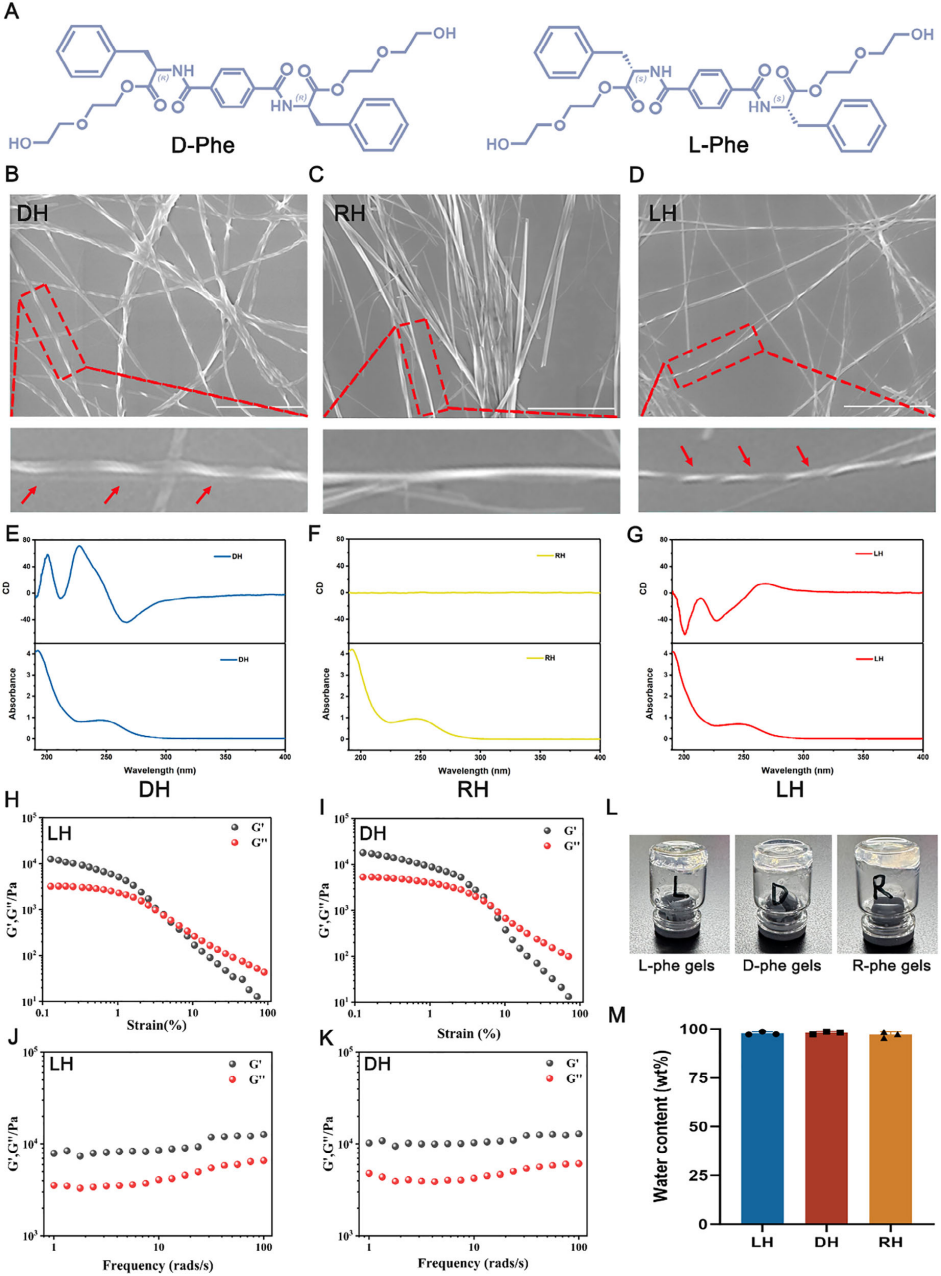
The characterization of chiral hydrogels. A) The chemical structure of L/D‐Phe. B–D) The representative SEM images of DH (Scale bar: 2 µm), RH (Scale bar: 5 µm) and LH (Scale bar: 2 µm) after freeze‐drying. E–G) The CD spectra and the corresponding UV/Vis spectra of DH, RH, and LH gels. H,I) Strain‐dependent oscillatory shear rheology of LH and DH hydrogels. J,K) Dynamic frequency sweep of LH and DH hydrogels. L) The photo images of L‐Phe, D‐Phe, and R‐Phe gels at room temperature. M) The water content of various chiral hydrogels (LH, DH, RH).

To investigate the effect of chiral hydrogels on LESCs, we selected biocompatible amino acid‐based gelators as building blocks to construct higher‐order chiral structures. Specifically, we utilized the two enantiomers of 1,4‐benzenedicarboxamide derivatives (D‐phe and L‐phe) to fabricate chiral hydrogels via a self‐assembly strategy (**Scheme** **1**; **Figure** **1**A). The chiral properties of these hydrogels were characterized using scanning electron microscopy (SEM) and circular dichroism (CD) spectroscopy. The LH and DH hydrogels exhibited distinct left‐handed and right‐handed helices, respectively, while the racemic mixture (RH) lacked discernible helical features (Figure 1B–D). Furthermore, the CD signals of the LH and DH enantiomers showed comparable intensities but opposite chiralities (Figure 1E–G). The left‐handed (LH) and right‐handed (DH) matrices displayed a perfect mirror‐image relationship, with increased CD signal intensities and spectral peaks at 200 and 225 nm, respectively (positive for DH and negative for LH). In contrast, the racemic mixture (RH), prepared by mixing D‐phe and L‐phe in a 1:1 molar ratio, exhibited no CD signal. These findings suggest that the chirality of these hydrogels arises from the self‐assembled fibrous aggregates rather than individual monomers.

In addition, the mechanical properties of supramolecular 3D‐chiral hydrogels (LH, DH, RH) were tested by a rotational rheometer with strain‐dependent oscillatory shear rheology and dynamic frequency sweep. The results demonstrated that the storage modulus (G’) of all three chiral hydrogels was higher than the loss modulus (G’’) with fixed strain, suggesting that these three supramolecular self‐assembled chiral hydrogels formed solid hydrogels (Figure 1H–K; Figure S1, Supporting Information). Furthermore, three chiral hydrogels (LH, DH, RH) all possess a certain degree of transparency (Figure 1L) and the water content of three chiral hydrogels were almost above 95% by the method of drying and weighing (Figure 1M). At the same time, the certain swelling property of chiral hydrogels can effectively adapt to the tear secretion of the ocular surface (Figure S2, Supporting Information). Then, we further examined the residence time of the chiral hydrogel on the ocular surface by performing anterior segment‐optical coherence tomography images. Although the retention time of chiral hydrogel was affected by blink and tear removal, the presence of chiral hydrogel could still be detected after 1.5 h (Figure S3, Supporting Information).

At present, hyaluronic acid (HA) gel has been widely used in the repair of ocular surface due to its unique biocompatibility, lubricity and water retention.^[^ ^50^, ^51^ ^]^ For example, Gong et al. coated the NGF‐loaded HA (10 w/v%) on 3D‐PCL microfibers scaffolds for conjunctival and ocular surface repair.^[^ ^52^ ^]^ Here, we first compared the mechanical properties of 10% HA gels (Figure S4, Supporting Information) and chiral hydrogels (Figure 1H–K; Figure S1, Supporting Information). Under a fixed strain of 0.5%, the storage modulus (G’) of DH consistently exceeds its loss modulus (G’’), indicative of a predominantly solid‐like gel behavior. In contrast, HA exhibits frequency‐dependent viscoelasticity, transitioning from a liquid‐like to a solid‐like state as the frequency increases. Under the frequency of 1 Hz, DH undergoes a strain‐dependent transition from solid‐like to liquid‐like behavior as strain increases, whereas HA remains in a liquid‐like state throughout the strain range. These results collectively highlight the superior mechanical strength and structural integrity of DH compared to HA. In addition, we further investigated the degradation rates of 10% HA gels and chiral hydrogels (DH) in vivo. The hydrogels mixed with cyanine 5.5 monoacid at a concentration of 0.001% (w/w) (0.2 mL) were injected into subcutaneous sites and fluorescence images of the cyanine‐containing gels were observed at different times (0, 1, 2, 3, 5 days). The results demonstrated that chiral hydrogels (DH) were almost undetectable after 5 days (Figure S5, Supporting Information) whereas 10% HA gels still preserved a certain amount of fluorescent signal (Figure S6, Supporting Information), indicating the faster degradation rates of DH compared with 10% HA gels in vivo. Oxygen permeability is a key parameter in the design of ocular surface materials, which may directly influence corneal metabolism, tear film stability, and the health of cornea. Here, we compared the oxygen permeability of 10% HA gels and chiral hydrogels (DH) and the results demonstrated that the oxygen permeability of chiral hydrogels (DH) (309.07/[cm^3^/(m^2^·d·Pa)]) was greater than 10% HA gels (26.22/[cm^3^/(m^2^·d·Pa)]) (Figure S7, Supporting Information), which further displayed the unique advantages and wide application prospects of chiral hydrogels over conventional options on ocular surface.

### 2.2 Biocompatibility of Chiral Hydrogels In Vitro and In Vivo

**Figure 2:**
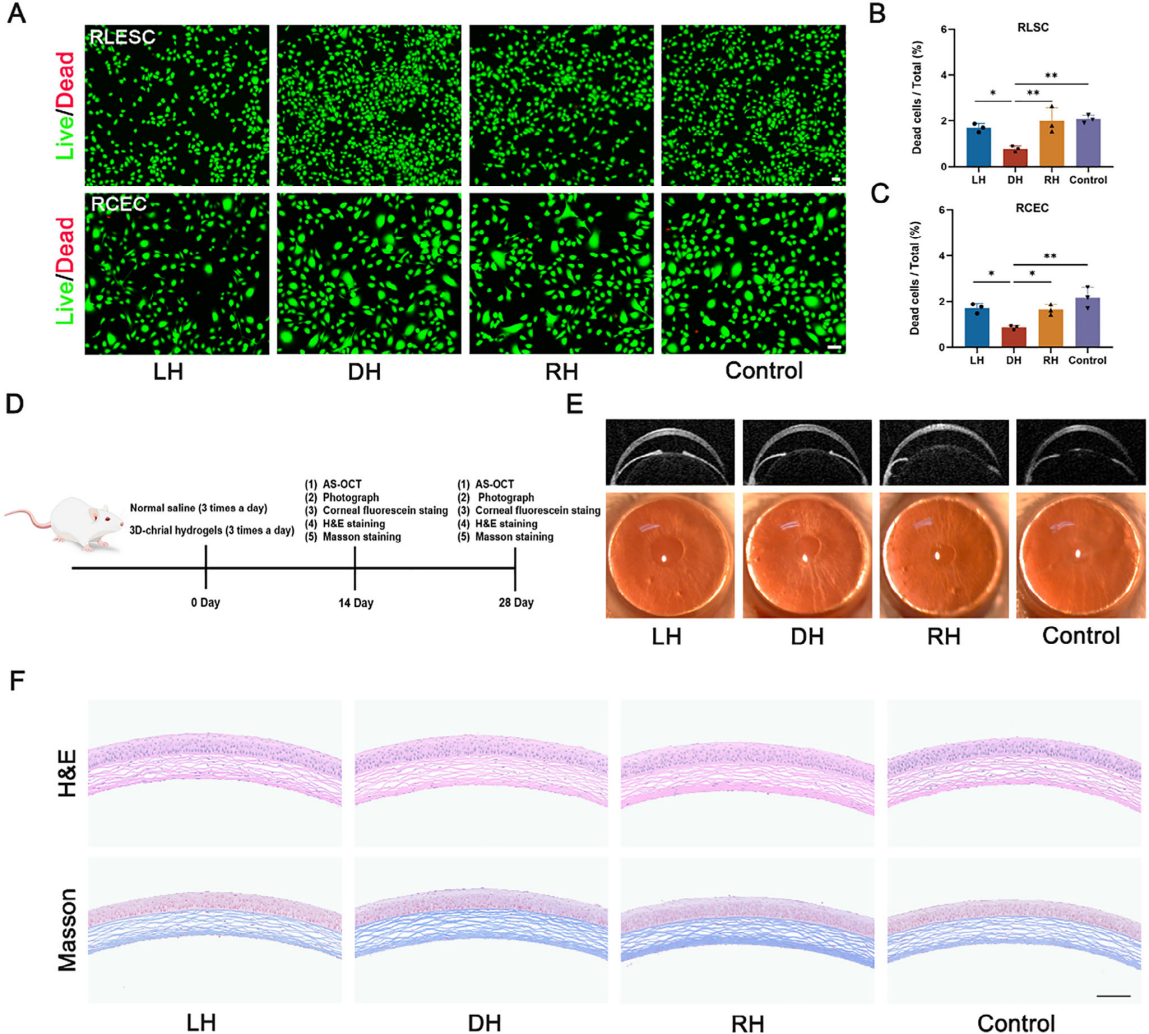
The biocompatibility of chiral hydrogels in vitro and vivo. A) The representative images of live/dead staining of LESCs and CECs at various chiral nanofiber matrixes. Scale bar: 50 µm. B,C) Statistical analysis demonstrated that DH presented the lowest cytotoxicity and better biocompatibility compared with other groups in LESCs and CECs. D) Schematic diagram of the investigation of in vivo cytotoxicity of chiral hydrogels. AS‐OCT: Anterior Segment‐Optical Coherence Tomography. E) Representative anterior segment‐optical coherence tomography and slit‐lamp microscope images after 14 days of normal saline or 3D‐chiral hydrogels treatments. F) Representative H&E and Masson staining images of corneas among various groups for 14 days. Scale bar: 100 µm. (n ≥ 3, *P < 0.05, **P < 0.01).

The biocompatibility is a crucial characteristic of tissue engineering materials. To evaluate the biocompatibility of chiral hydrogels, LESCs and CECs were cultured on the chiral nanofiber matrixes for 48 h and live/dead assays were performed to assess the cytotoxicity. As implied in **Figure** **2**A–C, the cell survival rates were more than 95% in all groups, and DH presented the lowest cytotoxicity compared with other groups in vitro. Figure. 2D illustrates the procedure of the in vivo investigation of chiral hydrogels. After topical administration of normal saline or 3D‐chiral hydrogels (LH, DH, and RH) for 14 and 28 days, the cornea of the mice with/without the hydrogel treatments all possessed great transparency and normal corneal appearance (Figure 2E; Figures S8 and S9, Supporting Information). Besides, to further investigate the potential toxicity, hematoxylin and eosin (H&E) and masson staining were also conducted to display the tissue structure. All experimental groups demonstrated a multilayered corneal epithelium and well‐organized stromal lamellae, with no signs of inflammatory cells infiltration, morphological abnormalities, or pathological lesions (Figure 2F; Figure S10, Supporting Information). Furthermore, after topical administration of chiral hydrogels for 14 and 28 days, the toxicity of chiral hydrogels to vital organs in balb/c mice was evaluated. H&E staining of major organs (heart, liver, spleen, lung and kidney) emerged with no obvious cell invasion, toxicity, inflammatory responses, or pathological responses compared with those in the control group, showcasing low toxicity and side effects in vivo (Figures S11 and S12, Supporting Information). In addition, we injected with the 3D‐chiral hydrogels (LH, DH, and RH) subcutaneously to further evaluate the potential toxicity of chiral hydrogels. The H&E staining of injected sites demonstrated complete stratification and normal morphological features with no abnormal changes at 14 days (Figure S13, Supporting Information) and 28 days (Figure S15, Supporting Information). Besides, the immunofluorescence staining of F4/80 and IL‐1β further presented the low immunogenicity and inflammatory response of chiral hydrogels in vivo at 14 days (Figure S14, Supporting Information) and 28 days (Figure S16, Supporting Information). Taken together, both in vitro and in vivo assessments disclosed the excellent biocompatibility and low cytotoxicity of chiral hydrogels to cornea and vital organs.

### 2.3 The Effects of Chirality on Proliferation and Migration of LESCs

**Figure 3:**
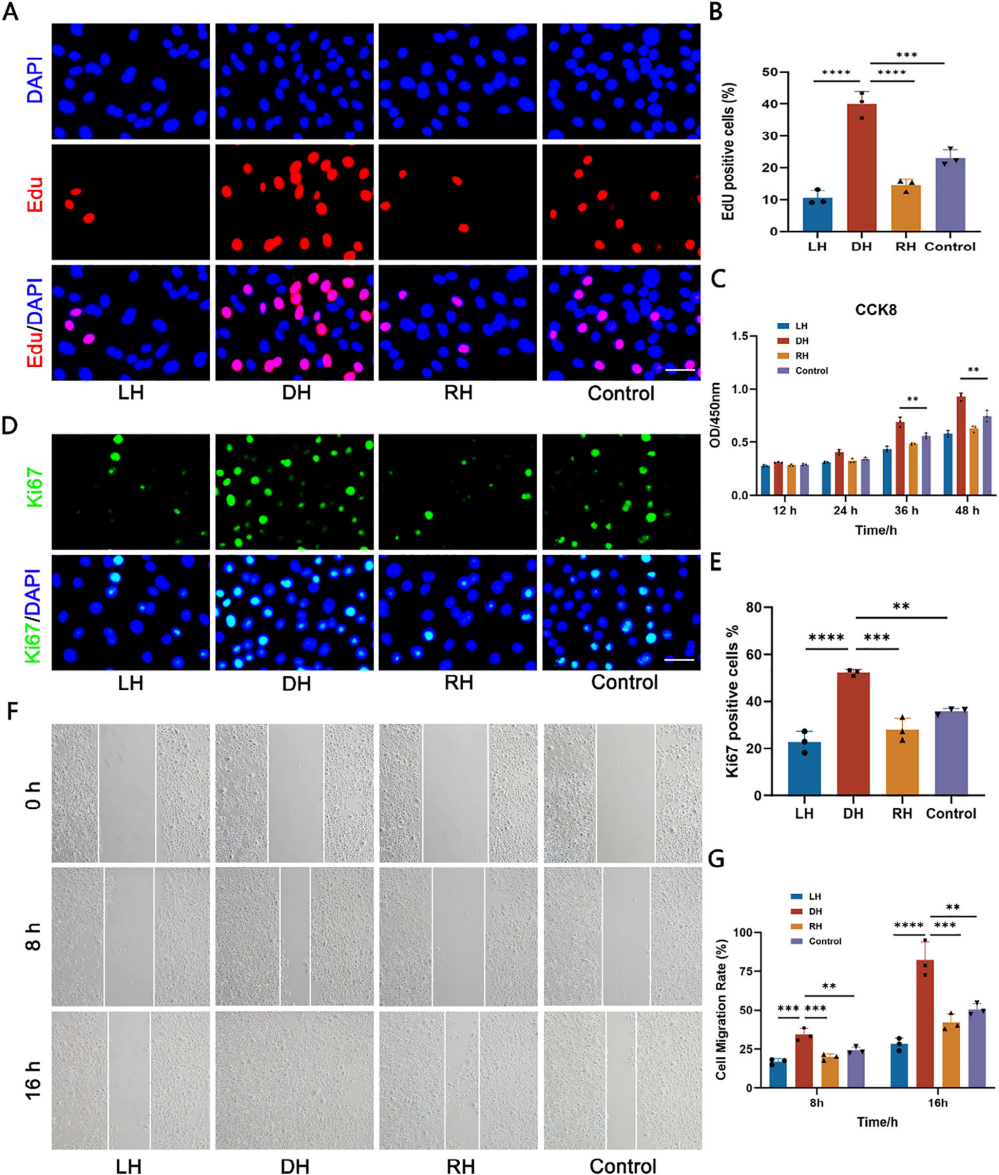
DH promoted the proliferation and migration of LESCs. A) The representative images of EdU staining at various chiral matrixes. Scale bar: 50 µm. B) Statistical analysis demonstrated higher EdU positive cells in DH group. C) The proliferation ability of LESCs cultured on various chiral matrixes was evaluated by CCK‐8 assay. D) The immunofluorescent staining of Ki67 among various groups. Scale bar: 50 µm. E) Statistic analysis of Ki67 positive cells. F) The migration capacity of LESCs cultured on various chiral matrixes was evaluated by the cell scratch assay at 0, 8, and 16 h. G) Statistic analysis of relative areas of migrated LESCs in different groups. (n ≥ 3, **P < 0.01, ***P < 0.001, ****P < 0.0001).

Since the capacity to promote cell proliferation, migration is a valuable characteristic of a cell carrier, especially in LSCD, we first identified primary rabbit LESCs (Figures S17 and S18, Supporting Information) and evaluated the influence of chirality on the proliferation capacity of LESCs using 5‐ethynyl‐2′‐deoxyuridine (EdU) incorporation staining and CCK‐8 assay. The cells with red fluorescence after EdU staining represented the occurrence of DNA replication and the proliferation ability of LESCs. It was observed that the proportion of LESCs proliferating on DH matrix (38.89%) was significantly higher than that in control group (23.07%) (**Figure** **3**A,B). Similarly, the CCK‐8 results demonstrated that there was a remarkable increase in the number of LESCs in the DH group compared to the LH, RH or control group after 12, 24, 36, and 48 h of co‐culture (Figure 3C). Furthermore, the immunofluorescence staining of proliferation marker Ki67 confirmed the capability of DH to significantly enhance the proliferation of LESCs (Figure 3D,E).

Subsequently, the cell migration assay was conducted to reveal the effect of chirality on the migration ability of LESCs (Figure 3F). It was observed that LESCs cultured on the DH matrix displayed faster migration rate than the control group by 10.81% in 8 h and 33.32% in 16 h (Figure 3G). The aforementioned results indicated that DH effectively promoted the proliferation and migration of LESCs.

### 2.4 DH Induced the Differentiation of LESCs, while LH Maintained the Stemness

**Figure 4:**
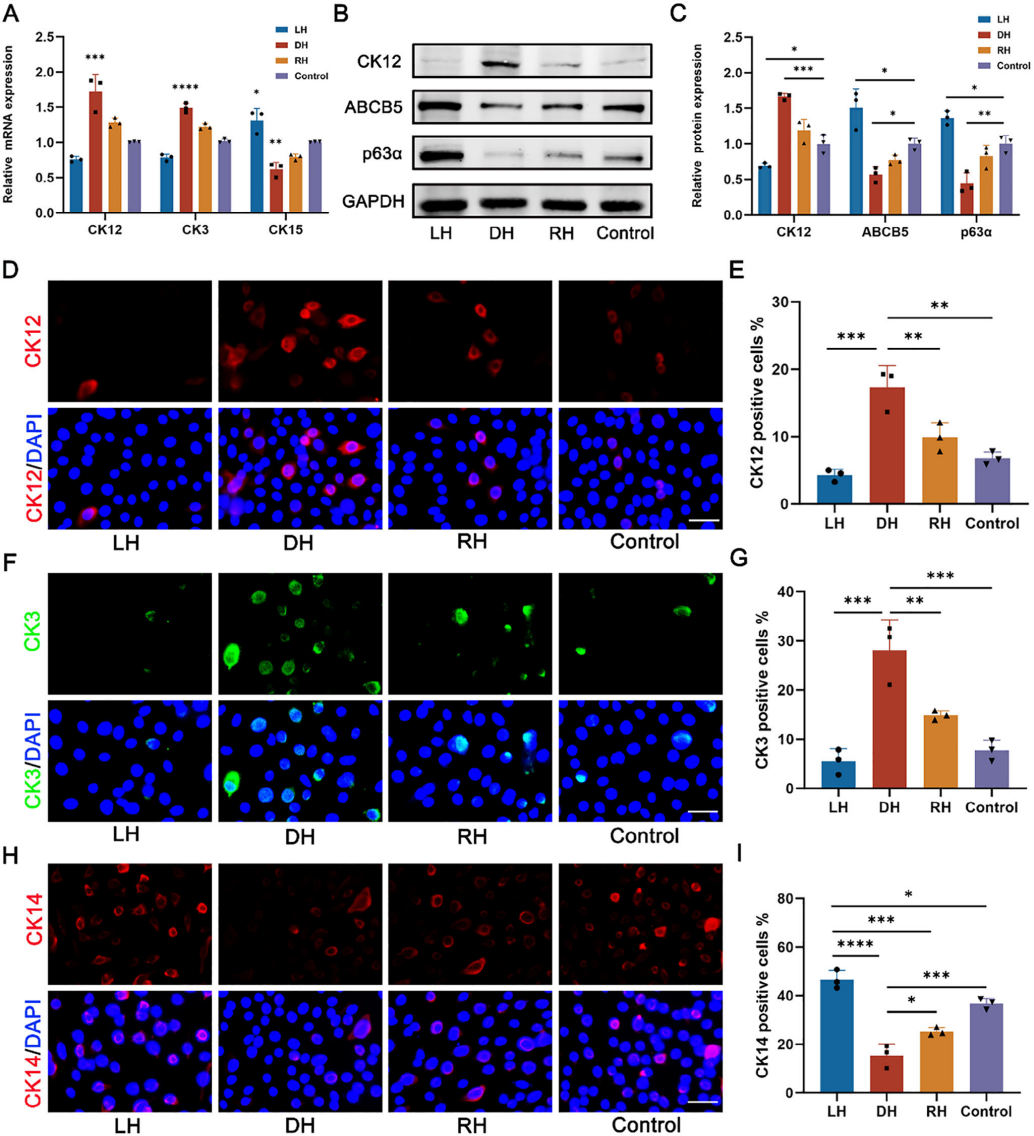
DH induced LESCs to differentiate into CECs, while LH maintained the stemness of LESCs. A) The qPCR assays revealed that differentiation‐related markers CK12 and CK3 were increased in the DH group, but stemness marker CK15 were decreased in the DH group. B) The protein expression of CK12, ABCB5 and p63α in various groups. C) Statistical analysis of relative protein expression levels of CK12, ABCB5, and p63α. D) Representative immunofluorescence staining images of CK12 at various chiral matrixes. Scale bar: 50 µm. E) Statistic analysis of positive cells of CK12 among various groups. F) The immunofluorescent staining of CK3 at various chiral matrixes. Scale bar: 50 µm. G) Statistic analysis of positive cells of CK3 among different groups. H) Representative immunofluorescence staining images of CK14 at various chiral matrixes. Scale bar: 50 µm. I) Statistic analysis of positive cells of CK14 among diverse groups. (n ≥ 3, *P < 0.05, **P < 0.01, ***P < 0.001, ****P < 0.0001).

To evaluate the impact of chiral microenvironment on the differentiation of LESCs, qPCR was conducted to reveal the regulatory effects of chiral hydrogels at the genetic level. The results showed that the differentiation markers CK12 and CK3 were up‐regulated in DH group, while the stemness marker CK15 was down‐regulated (**Figure** **4**A). Similarly, protein expression analysis using western blot demonstrated that CK12 was most highly expressed in the DH group, whereas the stemness markers ABCB5 and p63α were least expressed (Figure 4B,C). In contrast, the LH group exhibited opposite trends, with higher expression of stemness markers and lower expression of differentiation markers, suggesting that LH maintains LESCs stemness to some extent while DH promotes their differentiation into CECs.

To further confirm these findings, immunofluorescence staining was performed. Consistent with the above results, LESCs cultured on the DH matrix showed increased expression of CK12 and CK3, along with reduced expression of CK14 and p63α (Figure 4D–I; Figure S19, Supporting Information). Conversely, cells in the LH group exhibited elevated levels of CK14 and p63α. Collectively, these results indicate that DH promotes the differentiation of LESCs into CECs, while LH partially preserves the stemness of LESCs. Overall, we simultaneously revealed the opposite effects of two types of chiral hydrogels (LH and DH) on the differentiation and stemness maintenance of stem cells, which provide new insights to reveal the origin and development of organisms, elucidate the etiology of various diseases and promote tissue regeneration.

### 2.5 DH Accelerated the Corneal Epithelial Regeneration in Partial LSCD Models

**Figure 5:**
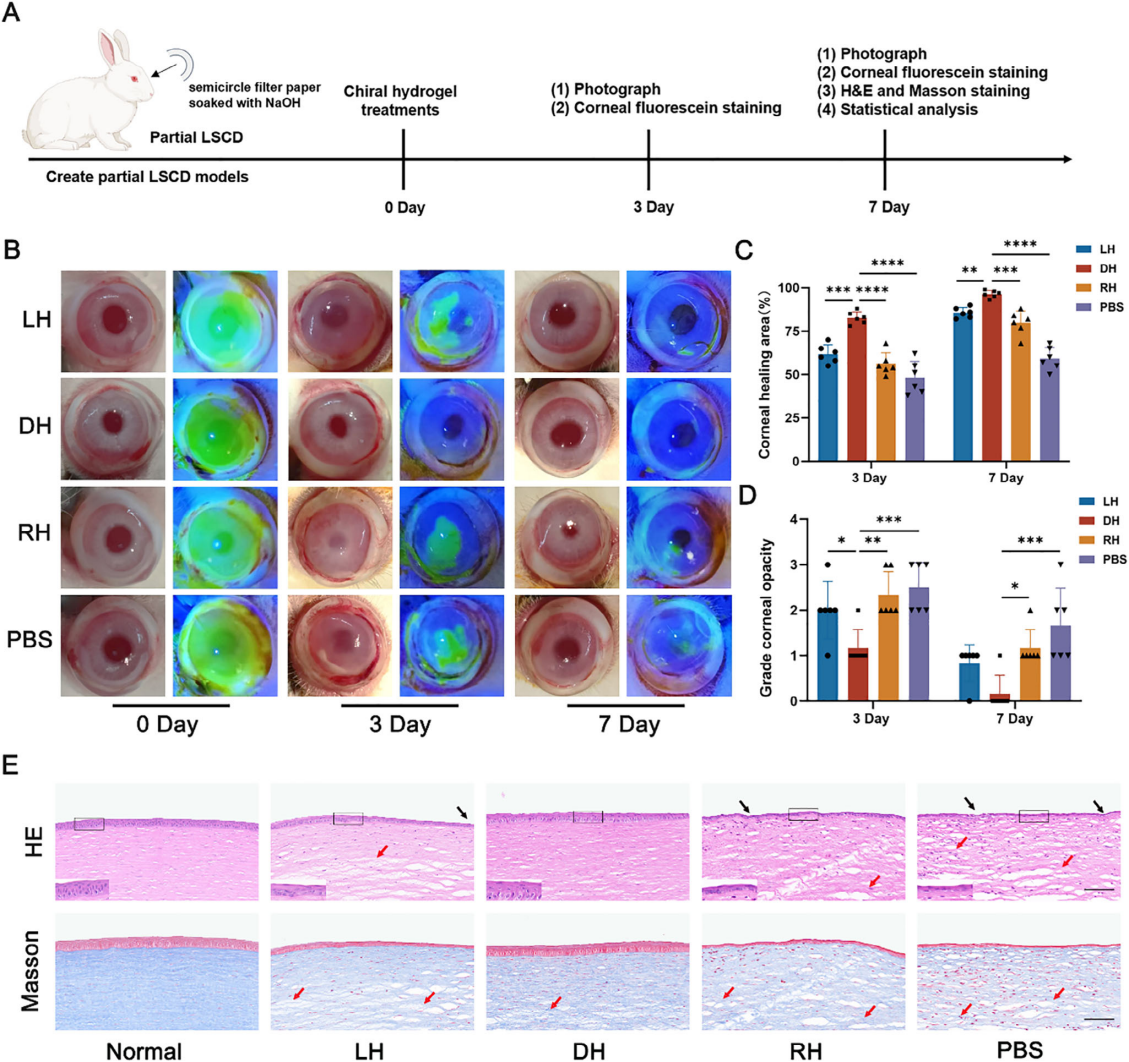
The therapeutic efficacy of DH in the treatment of partial LSCD rabbits. A) Flowchart for evaluating the therapeutic efficacy of chiral hydrogels in the partial LSCD rabbit models. B) Typical brightfield photos and fluorescein staining of rabbit eyes for in vivo evaluation on 0, 3 and 7 days post‐administration. C) Statistical analysis of repair area in each group on day 3 and 7. D) Statistical analysis of corneal opacity in each group on day 3 and 7. E) The representative H&E and Masson staining on day 7. Black arrows indicated corneal epithelial loss or damage, red arrows represented stromal edema and disorganized collagen arrangement. Scale bars: 100 µm. (n = 6, *P < 0.05, **P < 0.01, ***P < 0.001, ****P < 0.0001).

Based on in vitro experiments demonstrating that DH promoted the proliferation and migration of LESCs and induced their differentiation into CECs, we next investigated whether it would exert similar effects in vivo. Here, the partial LSCD rabbit models were first constructed by placing a semicircle Whatman III filter paper (breadth: 6 mm) presoaked with NaOH (Figure S20, Supporting Information) on the half limbus of the eye for 30 s and scraping all the central corneal epithelium (≈30–40 µm in depth). 3D‐chiral hydrogels were prepared by dissolving the enantiomers of a 1,4‐benzenedicarboxamide phenylalanine derivative molecules (L‐Phe, D‐Phe, or L‐Phe + D‐Phe) in dimethyl sulfoxide (DMSO). Then, ultrapure water was added to the resulting solution and then shaken up to form 3D‐chiral hydrogels (ultimate gelator concentration: 3.0 mg mL^−1^). Unlike traditional hydrogels, 3D‐chiral hydrogels have unique advantages of mimicking biophysical properties and architecture of natural ECM by a self‐assembly strategy to construct chiral microenvironment, thus manipulating cell behaviors effectively. Following, twenty‐four partial LSCD rabbits were randomly divided into four groups (six rabbits in each group): control group (treated with PBS three times a day), LH group (treated with LH three times a day), DH group (treated with DH three times a day) and RH group (treated with RH three times a day) (**Figure** **5**A). To visualize the injury of ocular surface, fluorescein was used to detect corneal epithelial defect. By Day 3, treated with the DH resulted in near‐complete corneal wound repair (82.67 ± 3.05%), significantly outperforming the other groups, where fluorescein staining remained detectable (corneal wound repair: LH: 61.83 ± 5.34%, RH: 56.17 ± 6.49%, PBS: 48.16 ± 9.41%) (Figure 5B). At 7 days after treatment, compared with other groups (LH: 85.67 ± 3.01%, RH: 79.83 ± 6.88%, PBS: 59.19 ± 6.62%), treated with DH emerged the faster closure of wounds (96.33 ± 2.16%) (Figure 5B,C). In addition, rabbits treated with DH demonstrated greater corneal transparency and less corneal vascularity, which were similar to normal rabbit cornea compared with other groups (Figure 5D).

In order to further assess the structural alteration after treatment, histological examination of the cornea was performed. H&E and masson stained corneal sections from rabbits with DH demonstrated multilayered epithelium and well‐organized stromal layer, which were comparable to normal cornea. In contrast, corneal sections in both PBS and RH‐treated groups displayed poor arrangement of epithelial cells, thinner epithelium, and irregular fibrosis, along with distinct neutrophil infiltration and edema in the stroma, which validated the superior therapeutic efficacy of DH (Figure 5E). In vivo studies also revealed a notable therapeutic effect of LH in partial LSCD models. This effect might be related to the ability of LH to enhance the stemness of LESCs. Under external stimulation, LH might support the maintenance of LESCs stemness and subsequently, a subset of these stem cells with enhanced stemness properties could be activated to differentiate into CECs, thereby facilitating corneal repair and achieving therapeutic benefits. However, the precise mechanisms underlying this phenomenon require further in‐depth investigation to elucidate the specific pathways involved.

Taken together, DH effectively promoted corneal epithelial regeneration in partial LSCD rabbit models. More importantly, the drug‐free property of DH eliminates concerns related to potential toxicity, rapid clearance, and unknown metabolic pathways associated with pharmaceutical interventions. This feature highlights its significant potential for broad applications and clinical translation, as well as meets the urgent need for innovative therapeutic strategies in the of treatment of LSCD and other corneal epithelium injury diseases.

### 2.6 DH Inhibited Inflammation and Vascularization both In Vitro and In Vivo

**Figure 6:**
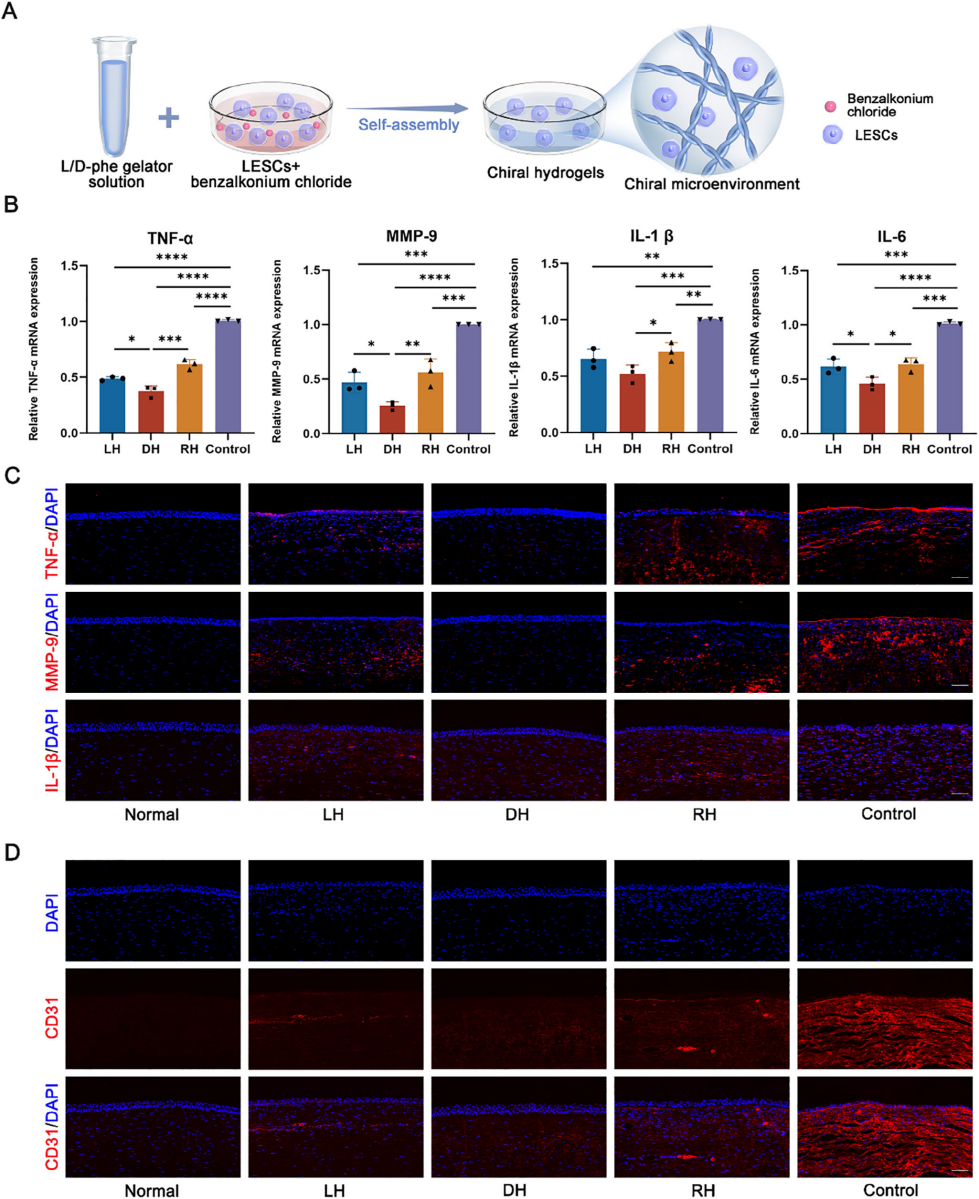
The inhibition of inflammation and vascularization in vitro and vivo by DH. A) Schematic diagram of evaluating the anti‐inflammation effect of chiral hydrogels in vitro. B) Statistical analysis of gene expression of inflammatory cytokines, including TNF‐α, MMP‐9, IL‐1β, and IL‐6 among various groups. C) The representative immunofluorescent staining images of TNF‐α, MMP‐9 and IL‐1β after different treatments. Scale bars: 100 µm. D) Representative immunofluorescent staining images of CD31. Scale bar: 100 µm. (n ≥ 3, *P < 0.05, **P < 0.01, ***P < 0.001, ****P < 0.0001).

Inflammation is one of the core pathogenesis of LSCD after trauma.^[^ ^19^, ^53^ ^]^ To evaluate the anti‐inflammation potential of chiral hydrogels, 0.0001% benzalkonium chloride was utilized to induce inflammatory injury to LESCs in vitro,^[^ ^54^ ^]^ followed by culturing them on different chiral matrixes for 48 h (**Figure** **6**A). qPCR was conducted to detect the expression of inflammatory cytokines, including tumor necrosis factor α (TNF‐α), matrix metalloprotein nine (MMP‐9), interleukin 1β (IL‐1β), and interleukin 6 (IL‐6). Compared to the control group, all chiral hydrogel‐treated groups (LH, DH, and RH) exhibited reduced expression of these inflammatory markers, with the D‐matrix chirality demonstrating the most pronounced inhibitory effect (Figure 6B). To further investigate whether this anti‐inflammatory response could be replicated in vivo, immunofluorescence staining was further performed to evaluate the inflammatory levels after various treatments. It was found that corneal alkali‐burn led to a significant increase of TNF‐α, MMP‐9 and IL‐1β expression in both the corneal epithelium and stroma. Notably, almost no red immunofluorescence was observed in the DH group, indicating its effective inhibition of the inflammatory response in contrast to other groups (Figure 6C). While LH and RH treatment also demonstrated a decrease in TNF‐α, MMP‐9, and IL‐1β immunoreactivity compared to the control group, their therapeutic efficacy was less pronounced than that observed in the DH group.

Furthermore, severe inflammation after acute alkali‐burn could drive pathological neovascularization.^[^ ^55^ ^]^ Here, the expression and distribution of vascular marker CD31 was investigated via immunofluorescence staining. Compared with control group, the growth of new capillaries appeared diminished or completely inhibited after DH treatment, demonstrating the potent anti‐angiogenic effect of DH in mitigating vascular proliferation (Figure 6D).

Taken together, DH not only effectively regulated the fate of LESCs and promoted corneal epithelial regeneration, but also played an important role in suppressing inflammation and neovascularization, thereby restoring a stable and homeostatic ocular surface microenvironment. Interestingly, previous studies have also reported the anti‐inflammatory effects of chiral hydrogels in macrophages and retinal progenitor cells,^[^ ^49^, ^56^ ^]^ further underscoring their promising therapeutic potential in ocular and regenerative medicine.

### 2.7 DH Regulated the Fate of LESCs via Activating Notch Signaling Pathway

**Figure 7:**
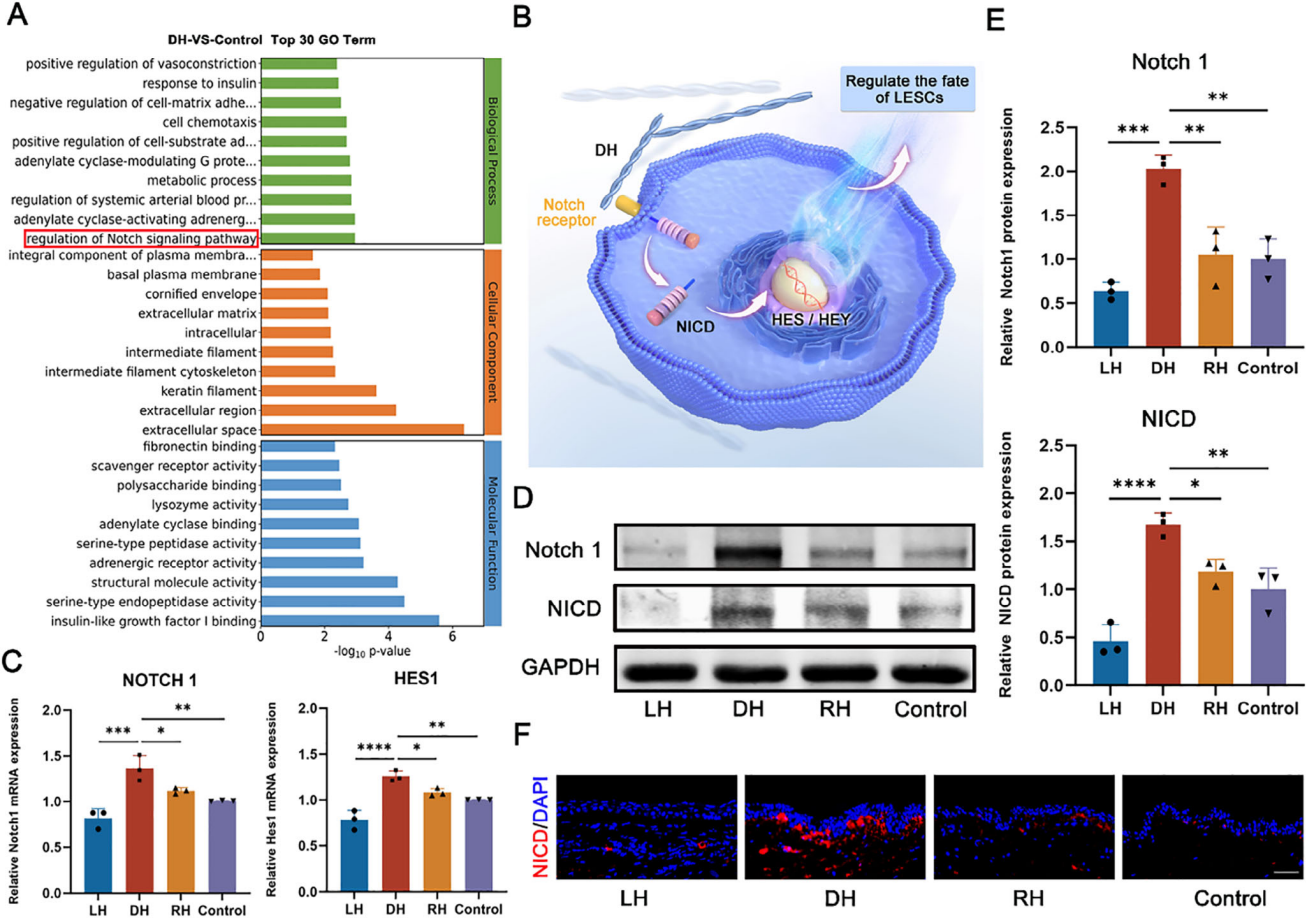
DH regulated the fate of LESCs through the activation of Notch pathway. A) The GO enrichment analysis of top 30 between DH versus control. B) Schematic representation of molecular mechanism in the fate of LESCs manipulated by DH. C) Quantitative RT‐qPCR analysis revealed Notch pathway related factors NOTCH1 and HES1 were upregulated in DH group. D) The protein expression of Notch1 and NICD among various groups. E) Statistical analysis of the relative protein expression levels of Notch1 and NICD. F) The immunofluorescence staining of NICD after various chiral hydrogels treatments. Scale bar: 50 µm. (n ≥ 3, *P < 0.05, **P < 0.01, *** *P* < 0.001, ****P < 0.0001).

Having demonstrated that chiral microenvironment played a crucial role in regulating LESCs fate commitment and tissue regeneration, we in‐depth investigated how the charity affected LESCs mechanistically. Here, we conducted pathway analysis of the obtained transcriptional profiles using Gene Ontology (GO) analysis (**Figure** **7**A). The findings revealed a significant upregulation of the Notch signaling pathway in the DH group, a pathway known to be critically involved in LESC fate commitment, differentiation, and the maintenance of corneal tissue homeostasis (Figure 7B).^[^ ^27^, ^57^, ^58^ ^]^ To verify the sequencing results, qPCR was conducted, confirming the upregulation of NOTCH1 and its downstream gene HES1(Figure 7C). These findings indicated that the Notch pathway was activated by the DH. Additionally, western blot analysis was performed, revealing a significant upregulation of key proteins related to Notch pathway, such as Notch1 and NICD, in the DH group (Figure 7D,E). Furthermore, LESCs cultured in the DH group were treated with the chemical inhibitor DAPT, which could block the Notch signaling pathway. As expected, gene expression of the Notch downstream target HES1 was markedly decreased in the DH+DAPT group. Notably, HES1 expression in the DH+DAPT group remained higher than that in the Control+DAPT group, further validating the critical role of the Notch pathway in mediating LESCs fate commitment by DH (Figure S21, Supporting Information). Subsequently, to further verify whether Notch pathway was activated during the process of corneal wound healing in vivo, the immunofluorescence staining was performed. As illustrated in Figure 7F, DH treatment resulted in significantly stronger NICD expression in basal LESCs compared to the other groups. This further underscores the pivotal role of the Notch signaling pathway in promoting corneal repair through DH. All these results proved that DH could effectively activate Notch pathway to enhance the proliferation and differentiation of LESCs, thereby promoted the corneal epithelial regeneration.

### 2.8 Molecular Dynamics Simulations Reveal Chirality Interaction Mechanisms

**Figure 8:**
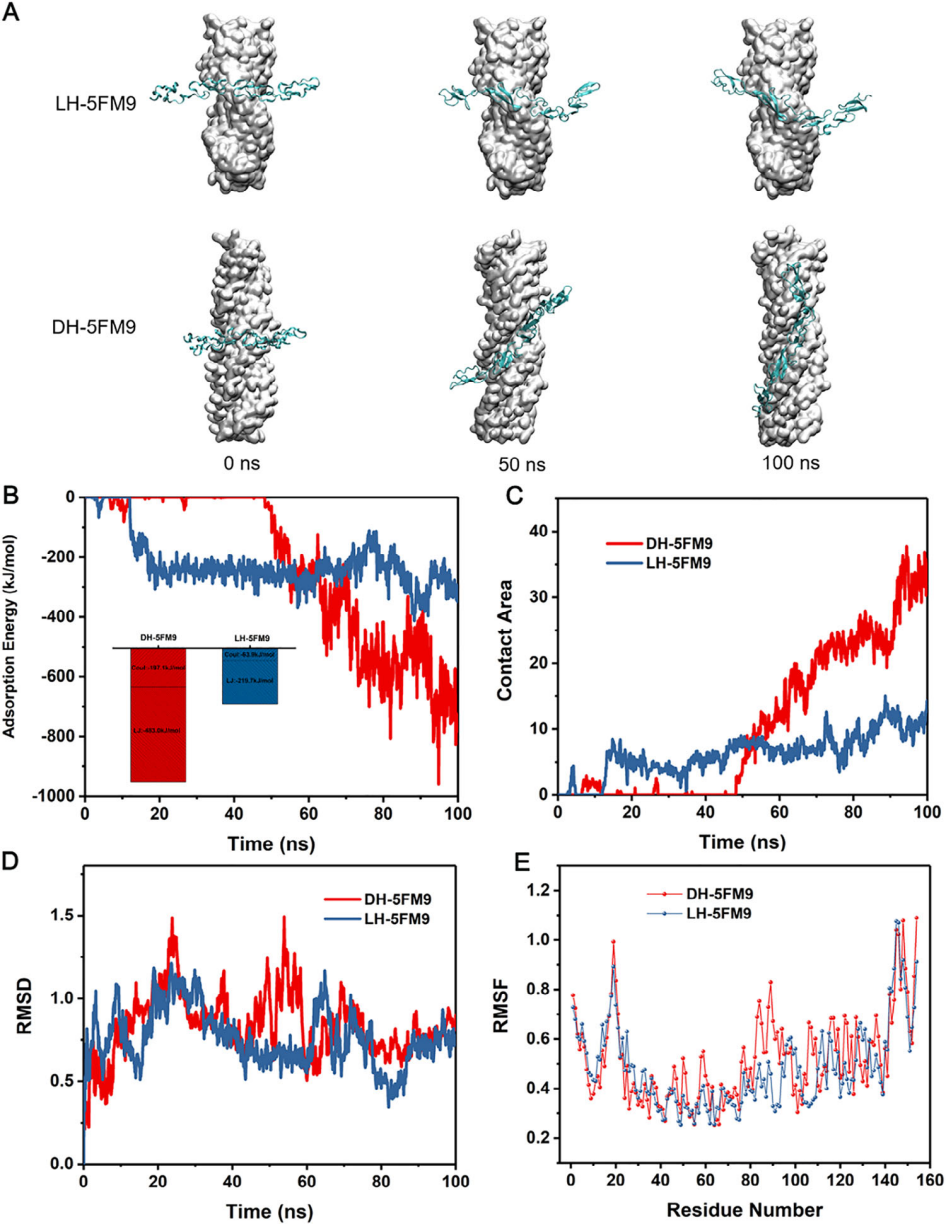
MD simulations proved the stereoselective interaction between 5FM9 and LH/DH. A) The snapshot of the adsorption of 5FM9 on LH/DH during the 100 ns MD simulations. B–E) The protein adsorption of 5FM9 on LH/DH, B) the adsorption energy, C) contact area, D) the root mean square (RMSD) and E) the root mean square fluctuation (RMSF) during the 100 ns MD simulations.

In order to gain a deeper understanding of the mechanisms of different chirality in regulating LESCs, classical molecular dynamic (MD) simulations were performed. Based on the experimental observation of the specific high expression of the Notch1 receptor, we selected the extracellular domain of the Notch1 receptor (RCSB: 5FM9) and employed MD simulations to investigate its selective recognition of chiral fibers. Simulation snapshots demonstrated that 5FM9 bound to the “protruding peak” of the LH fiber, while it adsorbed into the “groove” of the DH fiber. This enveloping adsorption exhibited higher binding energy (**Figure** **8**A). To elucidate the structural and property optimizations, we analyzed the components of the binding energy. The results indicated that van der Waals interactions dominated regardless of chirality. For the DH fiber, the van der Waals interactions reached ‐483 kJ mol^−1^, significantly surpassing other interaction forces (Illustration in Figure 8B). As shown in the contact area and root‐mean‐square deviation (RMSD) analyses (Figure 8C,D), 5FM9 adsorbed onto the LH fiber within a very short period, whereas it took 50 ns to adsorb onto the DH fiber, with stronger binding capacity (Figure 8D). Root‐mean‐square fluctuation (RMSF) analysis further revealed that more fluctuating amino acid residues were observed on the right‐handed fiber compared to the left‐handed fiber (Figure 8E). Taken together, the MD simulation data confirmed a greater stereo‐affinity interaction between DH and Notch1, ultimately leading to the activation of Notch signaling pathway, providing the basis for understanding the microenvironment of stem cell and directing biogenesis and regeneration.

It is undeniable that there are some potential confounding variables, including matrix stiffness and surface topography, and other signal pathways which may be involved in the fate regulation of LESCs by chirality. Further studies to deeply explore these factors on regulating the fate of LESCs will be conducted in the future. Overall, this study introduces chirality as a previously unknown cue for specifically manipulating LESCs behaviors, and provide new insights into chiral hydrogels to act as a drug‐free, effective approach for corneal epithelial regeneration, and offer a new direction for regenerative medicine and tissue engineering.

## 3 Conclusion

This study represents the first exploration of how a chiral microenvironment influences the fate of LESCs. Our findings reveal that DH not only enhances the proliferation and migration of LESCs, but also directs their differentiation into corneal epithelial cells. In contrast, LH contributes to partially preserve the stemness of LESCs, providing valuable insights into the role of chirality in stem cell biology and tissue regeneration. Inspired by these, we innovatively constructed 3D‐chiral hydrogels and demonstrated the therapeutic potential of DH in facilitating corneal epithelial regeneration, suppressing inflammation and neovascularization in LSCD rabbit models. Mechanistically, the fate determination of LESCs was driven by the activation of the Notch signaling pathway, reinforcing its pivotal role in stem cell differentiation and corneal homeostasis. Collectively, this study advances our understanding of LESCs fate regulation and corneal epithelial regeneration, highlighting the broader implications of stem cell microenvironment modulation. These findings pave the way for further stem cell and biomaterials research, and hold significant translational potential for the treatment of LSCD and other ocular surface diseases.

## Conflict of Interest

The authors declare no conflict of interest.

## Author Contributions

S.L., Y.Z., and J.C. contributed equally to this work. S.L., Y.Z., J.C., and L.C. drafted the manuscript and created all the figures. B.W., N.W., and H.S. examined the data. S.L., Y.Z., J.C., L.C., C.F., and Y.F. discussed the concepts of the manuscript. All authors contributed to the article and endorsed the final version of the manuscript.

## Supporting information



Supporting Information

## Data Availability

The data that support the findings of this study are available from the corresponding author upon reasonable request.
