# Supplementary material for: Chirality Regulates Stem Cell Fate and Promotes Corneal Epithelial Regeneration via Manipulating Notch Pathway
Source: Adv Sci (Weinh). 2025 May 30;12(31):e04732. doi: 10.1002/advs.202504732 (PMC12376501; doi:10.1002/advs.202504732)
Supplement: Supplementary file 1 — Supporting Information [file ADVS-12-e04732-s001.docx]

**Supporting Information**

**Chirality Regulates Stem Cell Fate and Promotes** **Corneal Epithelial Regeneration via Manipulating Notch Pathway**

*Shiding Li**^1,2#^, Yu Zhao^3#^, Junzhao Chen^1,2#^, Beibei Wu^3^, Nianxuan Wu**^1,2^, Hao Sun ^1,2^, Liangbo Chen^1,2*^,* *Chuanliang Feng^3*^, Yao Fu**^1,2*^*

^1^Department of Ophthalmology, Ninth People’s Hospital, Shanghai Jiao Tong University School of Medicine, Shanghai, China

^2^Shanghai Key Laboratory of Orbital Diseases and Ocular Oncology, Shanghai, China

^3^State Key Lab of Metal Matrix Composites, School of Materials Science and Engineering, Shanghai Jiao Tong University, Shanghai, China

^#^ These authors contributed equally to this manuscript.

*Corresponding author: Liangbo Chen, Postal address: 639 Zhizaoju Rd, Shanghai, China, 200011; E-mail address: chenliangbo@sjtu.edu.cn.

Chuanliang Feng, Postal address: 800 Dongchuan Rd, Shanghai, China, 200240; E-mail address: clfeng@sjtu.edu.cn.

Yao Fu, Postal address: 639 Zhizaoju Rd, Shanghai, China, 200011; E-mail address: fuyao@sjtu.edu.cn.

**Experimental Section**

*Matrix synthesis and characterization：* D/L-phenylalanine methyl ester hydrochloride, 1,4-benzenedicarbonyl dichloride, and diglycol were purchased from Aladdin Chemicals. 1,4-benzenedicarbonyl dichloride (2.6 g, 13.0 mmol) in dry dichloromethane (DCM) (20 mL) was added dropwise to a solution of D-phenylalanine methyl ester hydrochloride (6.0 g, 26.1 mmol) and triethylamine (Et3N, 8.0 mL, 58.3 mmol) in dry DCM (100 mL). The solution was stirred at room temperature for 24 h. All solvents were evaporated under vacuum, and the residue was subsequently dissolved in ethanol (100 mL). After filtration, the undissolved substance was collected and dried to give the dimethyl ester of p-Ph (DPhe-OH)2 (5.3 g, 10.9 mmol, 84%). For the hydrolysis, aqueous NaOH (10 ml, 2.0 M) was added to a cooled (25 °C) suspension of the dimethyl ester of p-Ph (D-Phe-OH)2 (3.0 g, 6.14 mmol) in MeOH (20 mL). The mixture was slowly returned to room temperature and stirred for 24 h, and a clear solution was obtained. The solution was then acidified with 3.0 M HCl until a pH value no greater than 3.0 was achieved and a gel-like precipitate formed. The gel phase was filtered, washed with deionized water, and finally dried in a vacuum oven to yield p-Ph (D-Phe-OH)2 (2.6 g, 5.6 mmol, 91%). The overall yield of p-(D-Phe-OH)2 was 76%. The 1 H NMR (400 MHz, DMSO-d6, δ, ppm) results are as follows: δ = 3.01-3.20 (m, 4H, CH2), 4.57-4.63 (m, 2H, CH), 7.14-7.17 (t, J = 4.0, 8.0 Hz, 2H, Ar-H), 7.22-7.26 (t, 4H,J = 4.0, 8.0 Hz, ArH), 7.28-7.30 (d, J = 8.0 Hz, 2H, Ar-H), 7.81 (s, 4H, Ar-H), 8.79-8.81 (d,J = 8.0 Hz, 2H, CO-NH), and 12.77 (s, 2H, COOH). Similarly, p-Ph-(L-Phe-OH)2 was obtained as a white solid (2.8 g, 98% yield).

*Charity of nanofiber matrixes and hydrogel preparation:* For in vitro experiments, the enantiomers of a 1,4-benzenedicarboxamide phenylalanine derivative molecules (L-Phe, D-Phe, or L-Phe + D-Phe) were suspended in deionized water (1mg/mL). Subsequently, the solution was heated to 95 °C to form a clear solution and then coated in cell-cultured plates. The hydrogel was observed in the plate wells when the solution cooled to the room temperature within a few minutes. After placing in the oven at 37 °C for 12 h, the hydrogel was allowed to form a nanofiber matrix on each well to mimic the chiral microenvironment. For in vivo experiments, three-dimensional chiral hydrogels were prepared by dissolving the enantiomers of a 1,4-benzenedicarboxamide phenylalanine derivative molecules (L-Phe, D-Phe, or L-Phe + D-Phe) in dimethyl sulfoxide (DMSO, final DMSO concentration: 3.3%). Then, ultrapure water was added to the resulting solution and then shaken up to form three-dimensional chiral hydrogels (ultimate gelator concentration: 3.0 mg·mL^−1^) [1-3].

*Scanning Electron Microscopy (SEM):* SEM images were acquired using a TESCAN RISE-MAGNA Microscope. The samples were prepared by depositing assembly solutions onto silicon wafers, which were then coated with a thin layer of Au prior to measurement.

*Circular dichroism (CD) spectroscopy:* The CD kinetic spectra were collected on a JASCO J-1500 CD spectrometer with a bandwidth of 1.0 nm.

*Rheological assay*: The rheological behavior was analyzed by a rotational rheometer (HAAKE MARS40, Thermo Fisher company, USA). The influence of strain amplitude and frequency on storage modulus and loss modulus were measured. For strain scanning mode, the strain varied from 0.1 to 100 % and the constant shear frequency was fixed as 1 Hz. For the frequency scanning mode, the shear frequency changed logarithmically between 0.1 and 100 rad s^-1^ with the fixed strain of 0.5 %.

*Hydrogel disassembly in vivo:* The chiral hydrogels (DH) or 10% HA mixed with cyanine 5.5 monoacid at a concentration of 0.001% (w/w) (0.2 mL) were injected into dorsal subcutaneous sites of balb/c mice. At different times (0, 1, 2, 3, 5 days), the fluorescence images of the cyanine-containing gels/HA were observed using an in vivo imaging system (IVIS).

*Swelling behavior* *assay:* The prepared chiral hydrogels (LH, DH, RH) were placed on the upper chamber of the 12-well cross-well plate. Then, chambers were immersed in 1 mL artificial tear (sodium hyaluronate). At predetermined time points, the weight of the chiral hydrogels was recorded. The swelling ratio of chiral hydrogels were calculated by the formula: Swelling ratio (%) = (W_1_ − W_0_)/W_0_ × 100%. W_0_ was the initial weight of the chiral hydrogels, and W_1_ was the final weight of the chiral hydrogels.

*System Preparation and MD Simulations:* The simulated systems (18*18*18 nm3) consisted of the DH/LH fiber, protein (RCSB:5FM9) and approximately 185890 water molecules and counterions of Na+ or Cl−. The initial position of the protein and fiber was consistent. The systems were modeled by the Amber14SB force field for the amino acids and ions [4], and the TIP3P model for water molecules [5]. The electrostatic interactions were evaluated using a particle-mesh Ewald (PME) summation [6], with a real space cutoff of 14.0 Å, and the Lennard−Jones (LJ) nonbonded interactions were smoothly tapered to 0 when the two atoms were 14 Å from each other. The LINCS method [7] was also used as a constraint algorithm. Three-dimensional periodic boundary conditions were applied in the simulations, and all of the simulations were carried out with a time step of 2 fs. The systems were initially relaxed for 1 ns under the NVT ensemble via the V-rescale thermostat, respectively. The simulated temperature is 300K and no pressure coupling is applied. Each system was simulated for 100ns. The structure images were generated with VMD software [8].

*Isolation and culture of rabbit LESCs:* All animal procedures in this study were approved by the Medical Ethics Committee of Ninth People’s Hospital, Shanghai Jiao Tong University School of Medicine (SH9H-2020-A177-1 for mice experiments, SH9H-2022-A531-SB for rabbit experiments) and carried out in accordance with the Experimental Animal Ethics Committee. The obtained limbal ring tissue from rabbits was soaked in phosphate-buffered saline containing penicillin-streptomycin and incubated with 10 mg/mL Dispase II (Sigma-Aldrich) at 4℃ for 12 h. Then, the limbal ring tissue was removed with the underlying stroma and digested with Tryple (Gibco) at 37℃ for 5 min until separation into individual LESCs. The obtained single-cell suspensions were seeded and cultured with F12/DMEM (Gibco) supplemented with 5% fetal bovine serum, 1% penicillin/streptomycin (P/S), B27 (Gibco), Y27632 (Selleck), and EGF (Proteintech) at 37 °C in a 5% CO_2_ environment. The medium was changed every 2 days.

*Live-dead assay:* For qualitative cell viability assessment, LESCs were cultured in 24-well culture plates coated with LH, DH or RH nanofiber matrixes for 48h. Then, each well was incubated with the live/dead cell staining reagents (Invitrogen) at the concentration of 1:2000 for ethidium homodimer-1 and 1:10,000 for calcein AM at 37℃ for 20 min in the dark. The representative images were processed and analyzed by ImageJ software.

*EDU proliferation assay:* LESCs were cultured in 24-well culture plates coated with LH, DH or RH nanofiber matrixes for 3 days. Then, EdU reagent (Beyotime) was added at the ratio of 1:1000. After 2 h of the culture at 37℃, the cells were fixed with 4 % paraformaldehyde at room temperature for 15 min. Then, EdU-mixed staining reagent configured according to the instructions was added and incubated at room temperature for 30 min in the dark. After washing with PBS, LESCs were stained with DAPI at room temperature for 20 min and imaged under the same fluorescence microscope.

*Cell Counting Kit-8 assay:* LESCs were seeded in blank or LH, DH, RH-coated 96-well plates at a density of 3 **×**10^3^ cells per well under proliferation conditions and each subgroup had 4 replicates. After 12h, 24h, 36h and 48h of incubation, the CCK-8 reagent was incubated in the culture at 37℃ for 4 h. The absorbance at 450 nm was measured and statistically analyzed.

*Immunofluorescence staining:* LESCs were seeded on 24-well plates coated with LH, DH or RH nanofiber matrixes at a density of 4x10^4^ per well for 3 days. Then, samples fixed with 4 % PFA for 20 min were permeabilized with 0.3 % Triton X-100 (Sigma-Aldrich) in PBS for 15 min, and blocked with 5 % goat serum (Sigma-Aldrich) for 1 h at room temperature. Subsequently, primary antibodies against Ki67 (1:200 dilution, 27309-1 AP, Proteintech), CK12 (1:200dilution, 24789-1-AP, Proteintech), CK3 (1:200 dilution, ab77869, Abcam), CK14 (1:200 dilution, ab119695, Abcam) and p63α (1:200 dilution, 4892, Cell Signaling Technology) were used to stain the samples at 4℃ overnight. The next day, samples were incubated with Alexa Fluor-conjugated secondary antibodies (1:400 dilution) at room temperature for 1 h in the dark. Nuclei were stained with DAPI (Invitrogen) for 20 min and images were taken by fluorescent microscope.

*Cell migration assays:* To perform the cell migration assay, LESCs were seeded on 6-well plates coated with various chiral matrixes at a density of 2×10^5^ cells per well. When the cells reached 80–90% confluence, scratch wounds were scratched vertically using a 200 μL sterile pipette tip. After washing with PBS (Gibco) three times, the scratch wounds were imaged by digital micro scope at 0 h, 8 h and 16 h. The data were calculated by Image J software.

*Western blot analysis:* LESCs were seeded on 6-well plates coated with chiral matrixes at a density of 2 × 10^5^ cells per well for 3 days. For Western blot analysis, total protein lysates were extracted using RIPA Lysis Buffer (Beyotime). Samples were separated on a 10 % polyacrylamide gel and electrophoretically transferred to 0.22 μm PVDF membranes (Millipore). Next, the membranes were blocked with 5 % BSA in TBS buffer and incubated with primary antibodies against CK12 (1:1000 dilution, 24789-1-AP, Proteintech), ABCB5 (1:1000 dilution, sc-515910, Santa Cruz), p63α (1:1000 dilution, 4892, Cell Signaling Technology), Notch1 (1:1000 dilution, ab52627, Abcam), NICD (1:1000 dilution, 4147, Cell Signaling Technology) and GAPDH (1:5000 dilution, ab8245, Abcam) overnight at 4 ℃. The next day, the membranes were washed three times using Tris Buffered saline Tween (TBST) and incubated with fluorescent-conjugated secondary antibody for 1 h at room temperature and scanned.

*Total RNA Separation and quantitative polymerase chain reaction (qPCR):* The total RNA was extracted from LESCs seeded on 6-well culture plates coated with LH, DH or RH chiral matrixes with RIPA, and the harvested total RNA concentrations were detected by spectrophotometry using NanoDrop™ 2000 software (Thermo Fisher Scientific). Employing a high-capacity reverse transcription kit for RTPCR, cDNA was synthesized with 15 min at 95 ◦C, followed by 40 cycles of denaturation for 10 s at 95 ◦C and annealing at 60 ◦C for 30 s. The real-time PCR was carried out with SYBR Green Mix (Yeasen) to quantify the mean values of delta Ct and Standard Error of Mean. Primers sequences used in the research are presented in Table S1. Chemical inhibitor DAPT (10μM, ab120633, Abcam) was used to block the Notch signaling pathway.

*Construction of* *the partial LSCD rabbit models:* All animal procedures with rabbits in this study were approved by the Medical Ethics Committee of Ninth People’s Hospital, Shanghai Jiao Tong University School of Medicine and carried out in accordance with the Experimental Animal Ethics Committee. New Zealand 10-12 weeks white rabbits, weighing about 2 kg, were used in this study, and the partial LSCD rabbit models were established as described. In brief, rabbits were anesthetized by intramuscularly injecting Zoletil (Virbac S.A.) and received ocular administration of oxybuprocaine hydrochloride eye drops (Santen Pharmaceutical, Kita-ku, Osaka, Japan), then a semicircle Whatman III filter paper (breadth: 6mm) presoaked with 1 N NaOH (Sigma Aldrich) was placed on the half limbus of the right eye for 30 s, followed by flushing the wound surface with PBS for 1 min. Then, all the central corneal epithelium was scraped gently (epithelial scraping depth was about 30-40μm). The defect sites were stained with sodium fluorescein (Sigma Aldrich) and the defect repair was recorded by imaging.

*Corneal clarity evaluation:* The corneal clarity was graded as follows: grade 0, completely clear with no opacity observed; grade 1, haze of minimal density seen with difficulty on direct and diffuse illumination; grade 2, slightly hazy, iris and pupil are easy to see; grade 3, severely opaque iris with barely visible pupils; and grade 4, completely opaque, with no iris and/or pupil visible [9].

*Histology and immunofluorescence staining:* Cornea tissues were resected after the rabbits were euthanized. Then the tissues were fixed in 4% PFA and paraffin-embedded for sectioning. Representative sections were stained with H&E to evaluate the morphology of cornea epithelial, Masson trichrome to detect irregular fibrosis with collagen deposition. For immunofluorescence staining, the sections were blocked with 5% donkey serum in PBS for 1 h at room temperature and incubated with the primary antibody of TNF-α (1:1000 dilution, ab183218, Abcam), MMP-9 (1:1000 dilution, ab76003, Abcam), IL-1β (1:1000 dilution, ab254360, Abcam) and CD31(1:1000 dilution, ab9498, Abcam) at 4℃ overnight, following by incubating with the corresponding secondary antibody for 1 h at room temperature and stain nuclei with DAPI.

*Quantification and statistical analysis:* Statistical analyses were performed using GraphPad Prism10.0 for Windows. Data are presented as means ± standard deviation (SD). All experiments were performed at least 3 repeats. Two-sample equal variance t-test was and One-way ANOVA were used to analyze the significance. A P value < 0.05 indicated a statistically significant difference.

**References:**

1. Liu G, Zhang D, Feng C, *Angew Chem Int Ed Engl*. **2014**, *53*, 7789.
2. Yang L, Yang L, Lu K, Su N, Li X, Guo S, Xue S, Lian F, Feng C, *Adv Sci (Weinh)*. **2023**, *10*, e2304627.
3. Liu J, Zhao Y, Zhao C, Dou X, Ma X, Guan S, Jia Y, Feng C, *Nano Research*. **2022**, *15*, 2226.
4. Maier JA, Martinez C, Kasavajhala K, Wickstrom L, Hauser KE, Simmerling C, *J Chem Theory Comput*. **2015**, *11*, 3696.
5. Jorgensen, W. L.; Chandrasekhar, J.; Madura, J. D.; Impey, R. W.; Klein, M. L, *J. Chem. Phys.* **1983**, *79*, 926.
6. Darden, T.; York, D.; Pedersen, L, *J. Chem. Phys.***1993**, *98*, 10089.
7. Hess, B.; Bekker, H.; Berendsen, H. J. C.; Fraaije, J. G. E. M, *J. Comput. Chem.* **1997**, *18*, 1463.
8. Humphrey W, Dalke A, Schulten K, *J Mol Graph.* **1996**, *14*, 33.
9. Liu N, Zhang X, Li N, Zhou M, Zhang T, Li S, Cai X, Ji P, Lin Y, *Small*. **2019**,*15*, e1901907.

**Results and discussion**

**
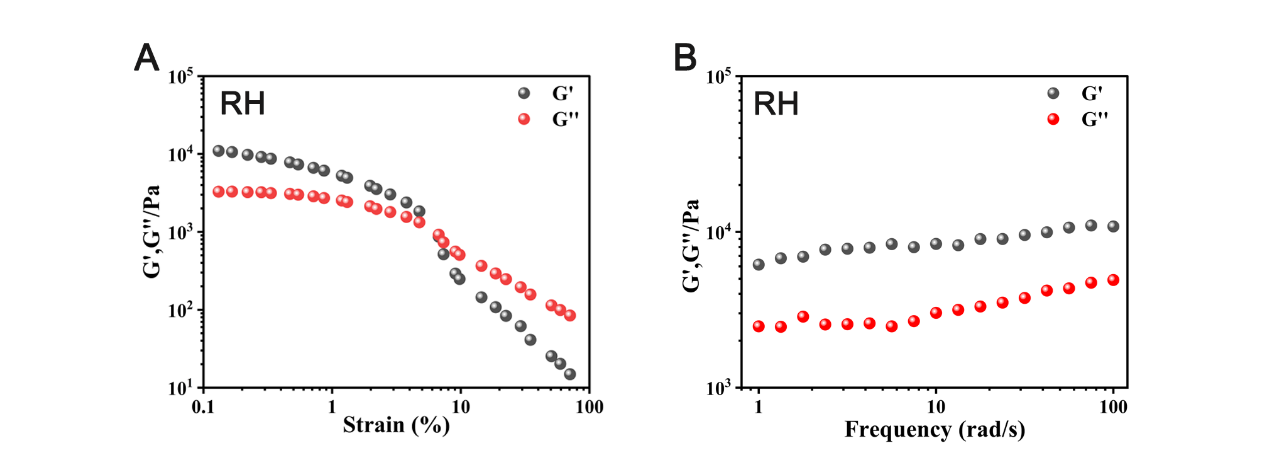
**

**Figure S1.** Strain-dependent oscillatory shear rheology (A) and Dynamic frequency sweep (B) of RH hydrogels.


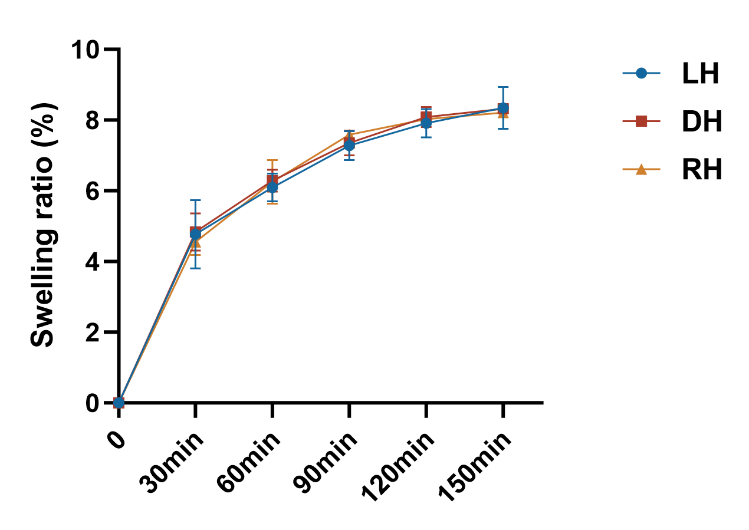


**Figure S2.** The swelling ratio of chiral hydrogels.


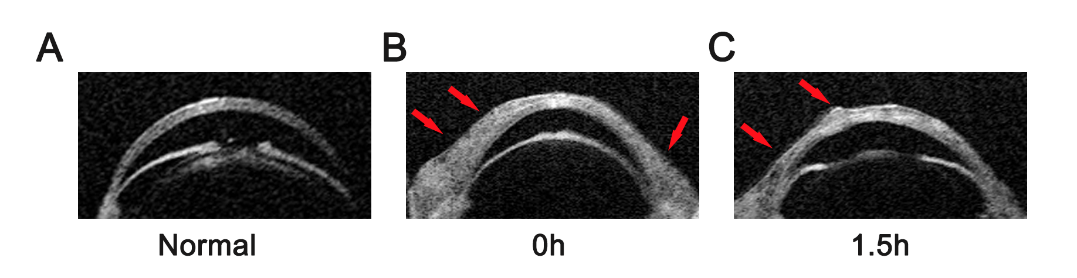


**Figure S3.** The representative AS-OCT images from mice eyes before (A) and after topical administration of chiral hydrogels at 0 h (B) and 1.5 hours (C).


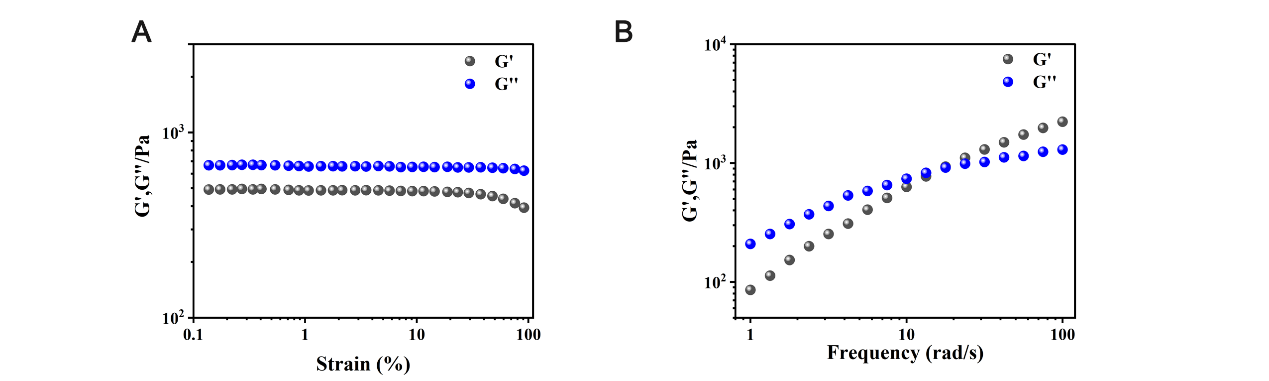


**Figure S4.** The strain-dependent oscillatory shear rheology (A) and dynamic frequency sweep (B) of 10% HA.


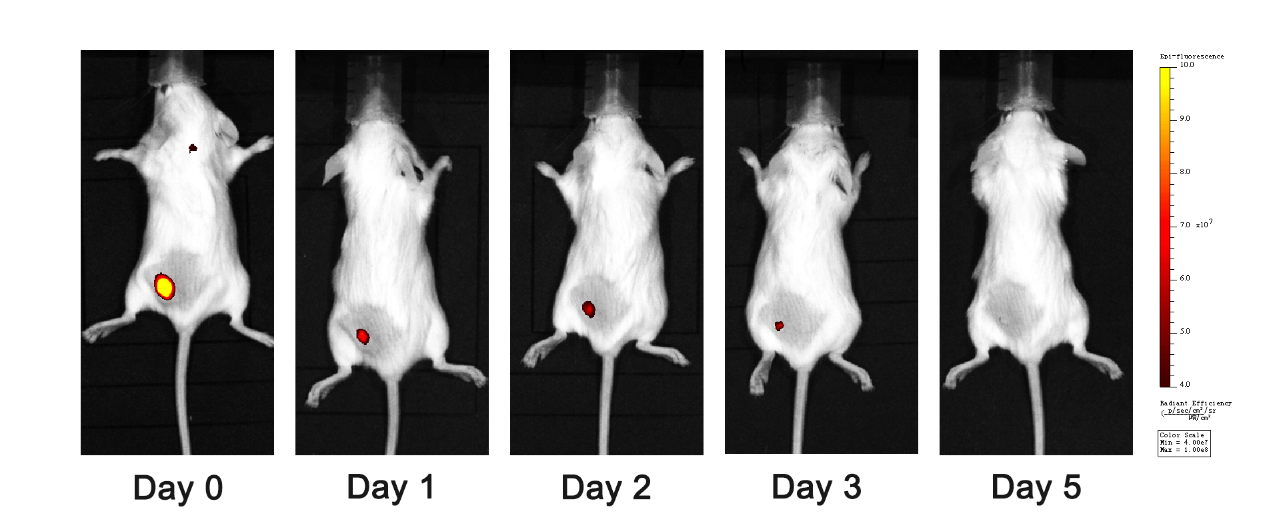


**Figure S5.** Representative in vivo imaging system images of DH-embedded cyanin.


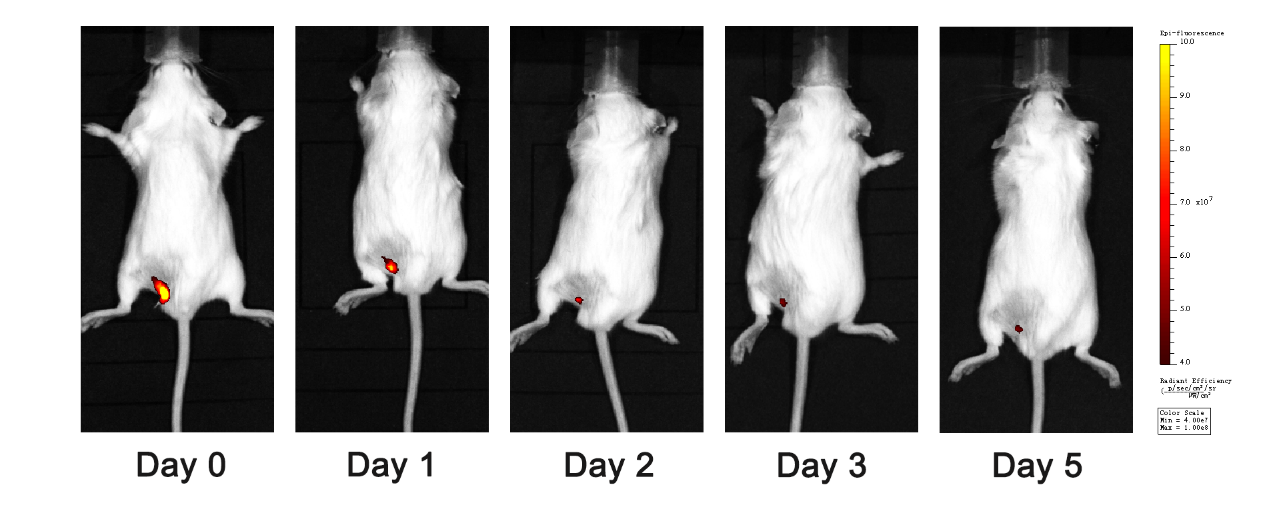


**Figure S6.** Representative in vivo imaging system images of 10 % HA-embedded cyanin.


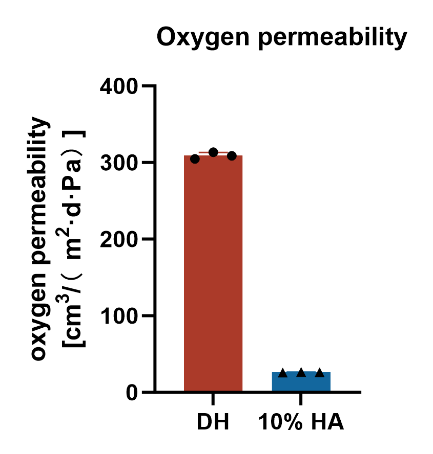


**Figure S7.** The oxygen permeability of DH and 10% HA.


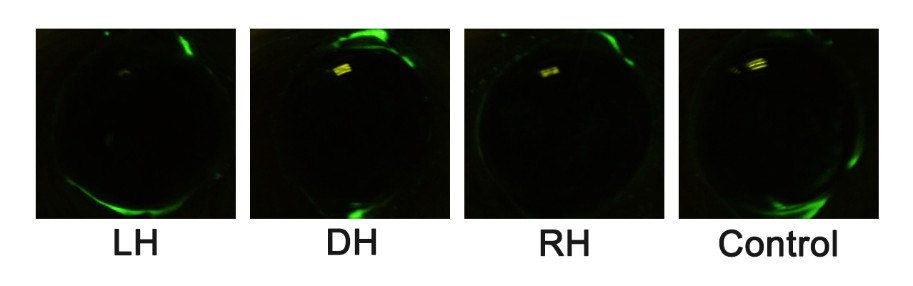


**Figure S8.** The corneal sodium fluorescein staining after 14 days of normal saline (control) or 3D-chiral hydrogels treatments.


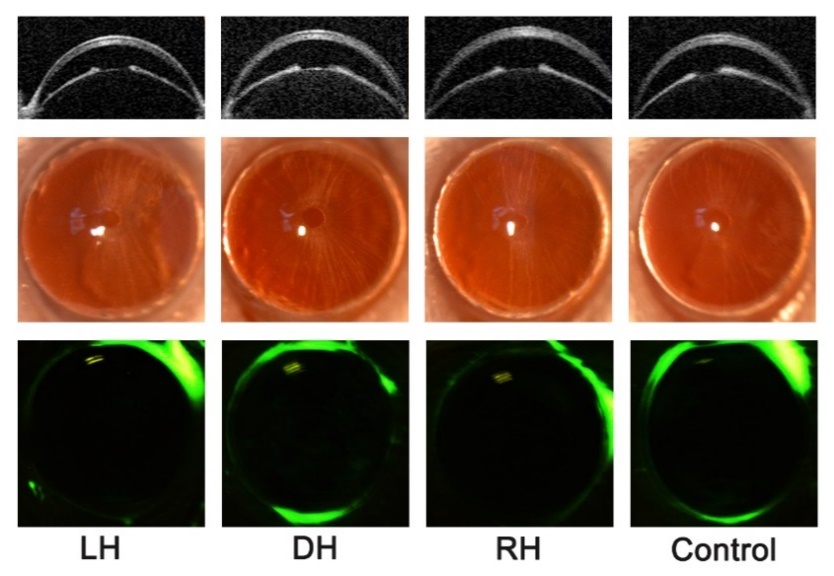


**Figure S9.** Representative anterior segment-optical coherence tomography, slit-lamp microscope images and corneal sodium fluorescein staining after 28 days of normal saline (control) or 3D-chiral hydrogels treatments.


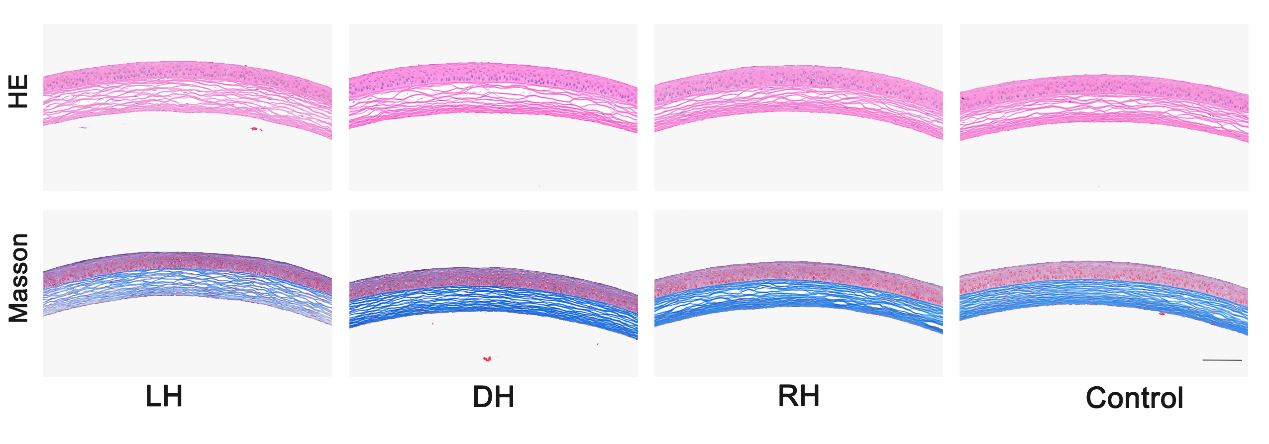


**Figure S10.** Representative H&E and Masson staining images of corneas among various groups for 28 days. Scale bar: 100 μm.


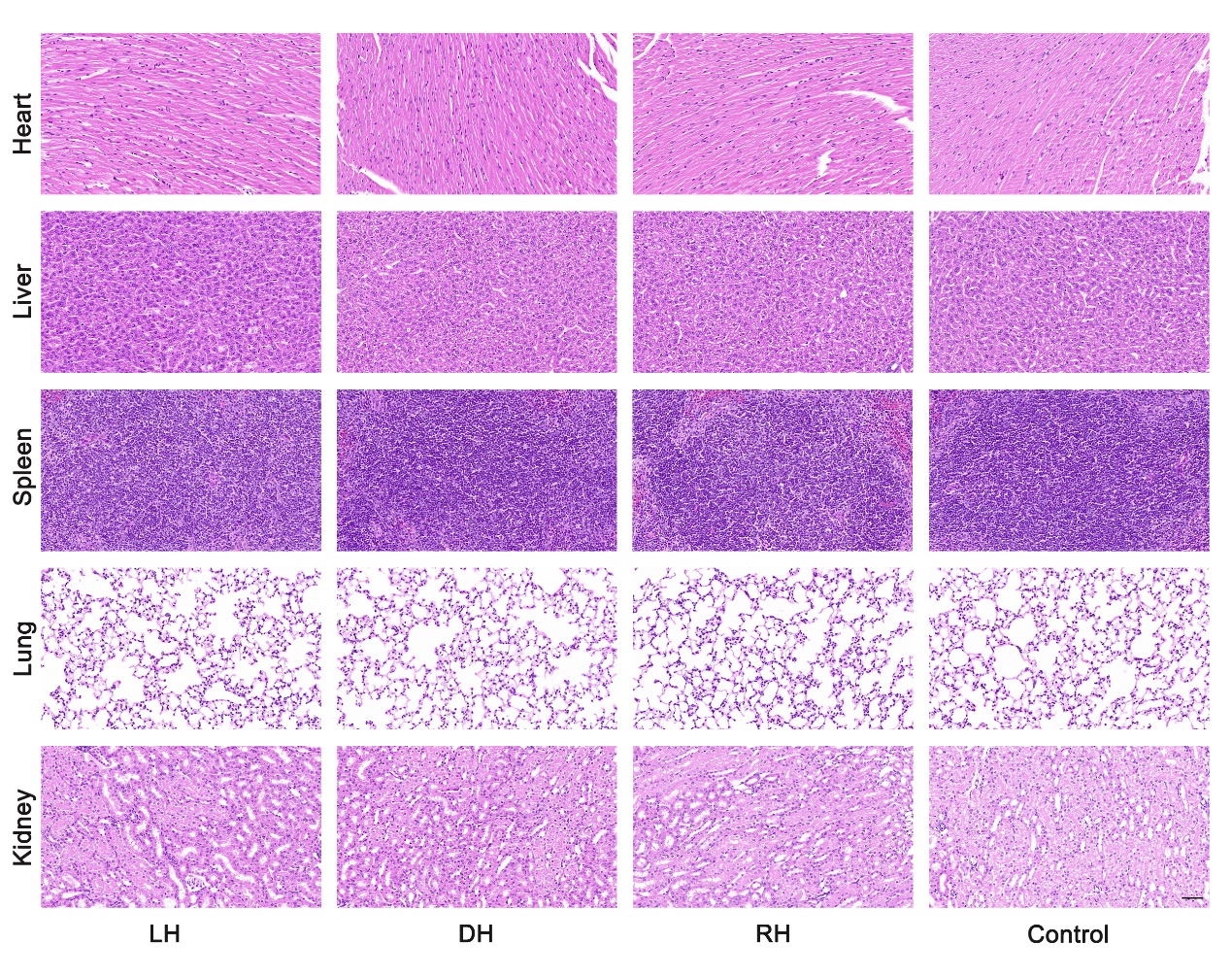


**Figure S11.** The representative histopathological examinations of the main organs (including heart, liver, spleen, lung, kidney) after topical administration of chiral hydrogels or normal saline (control) for 14 days via H&E staining. Scale bar: 50 μm.


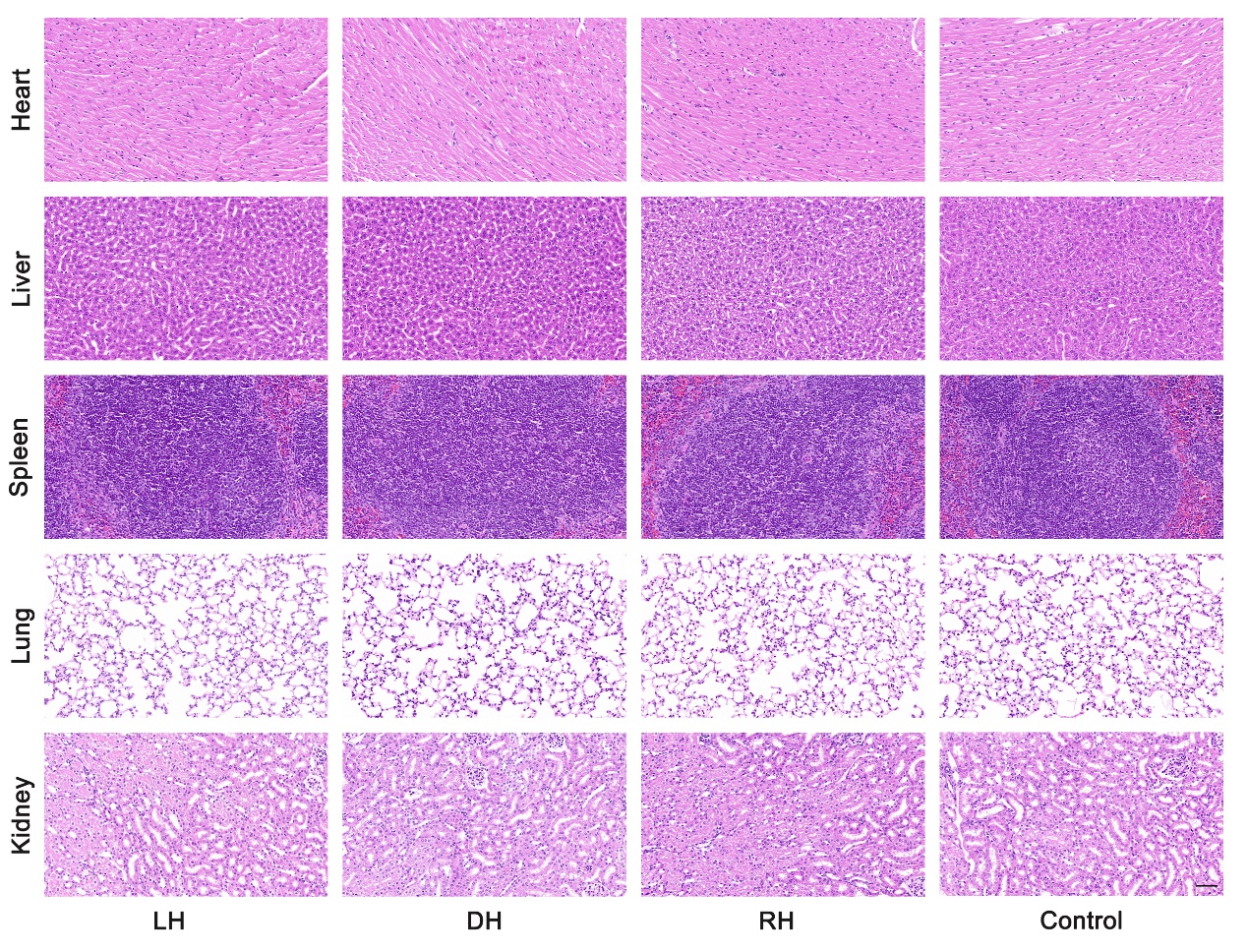


**Figure S12.** The representative histopathological examinations of the main organs (including heart, liver, spleen, lung, kidney) after topical administration of chiral hydrogels or normal saline (control) for 28 days via H&E staining. Scale bar: 50 μm.


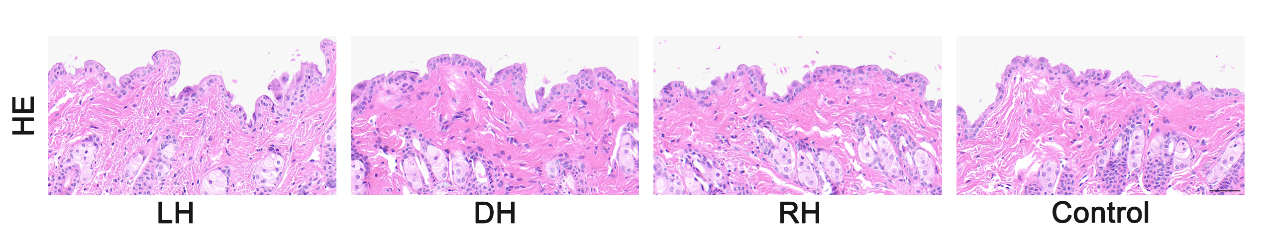


**Figure S13.** The representative H&E staining after subcutaneous injection of chiral hydrogels on day 14. Scale bar: 50 μm.


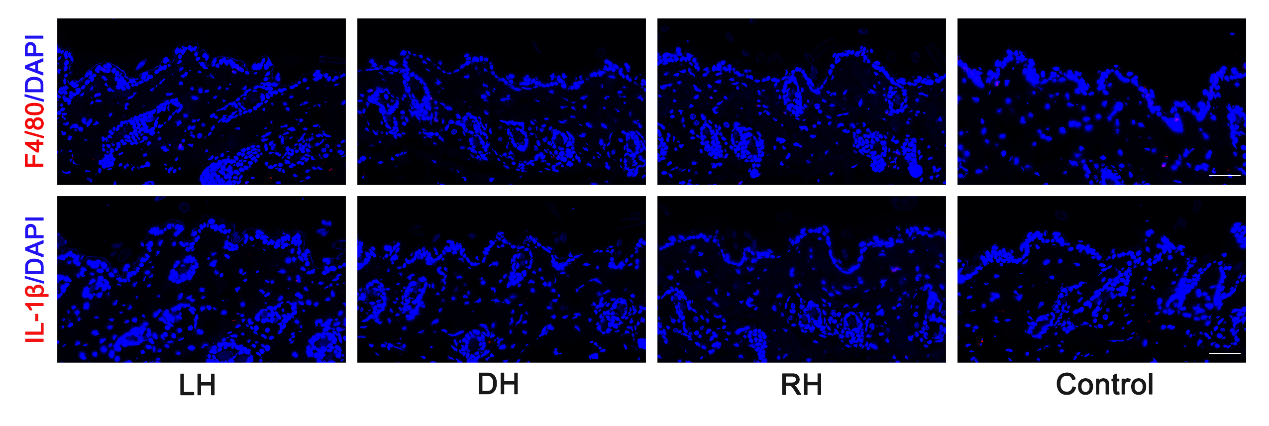


**Figure S14.** The immunofluorescence staining of F4/80 and IL-1β after subcutaneous injection of chiral hydrogels on day 14. Scale bars: 50 μm.


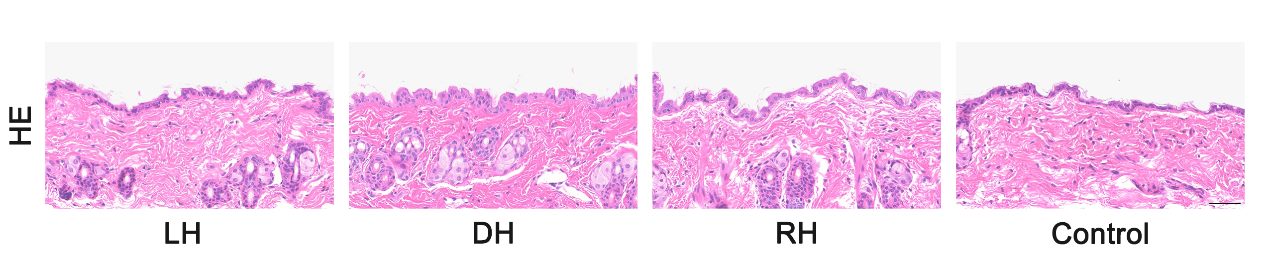


**Figure S15.** The representative H&E staining after subcutaneous injection of chiral hydrogels on day 28. Scale bar: 50 μm.


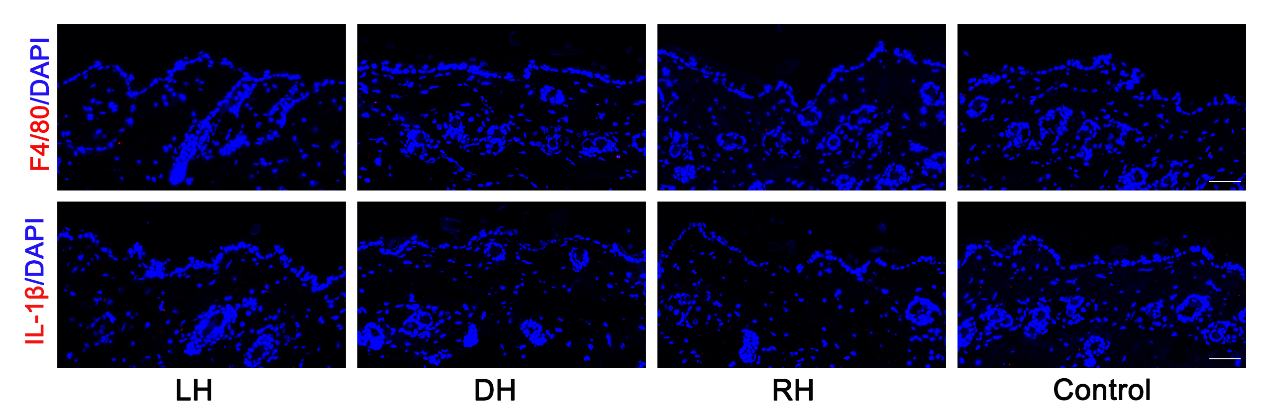


**Figure S16.** The immunofluorescence staining of F4/80 and IL-1β after subcutaneous injection of chiral hydrogels on day 28. Scale bars: 50 μm.


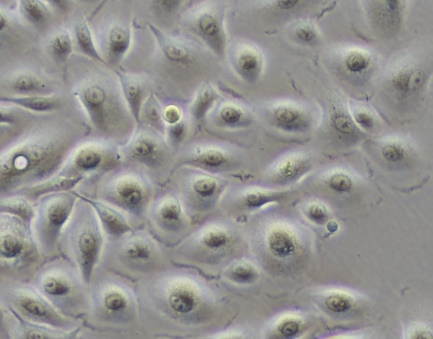


**Figure S17.** The bright-filed images of primary rabbit LESCs


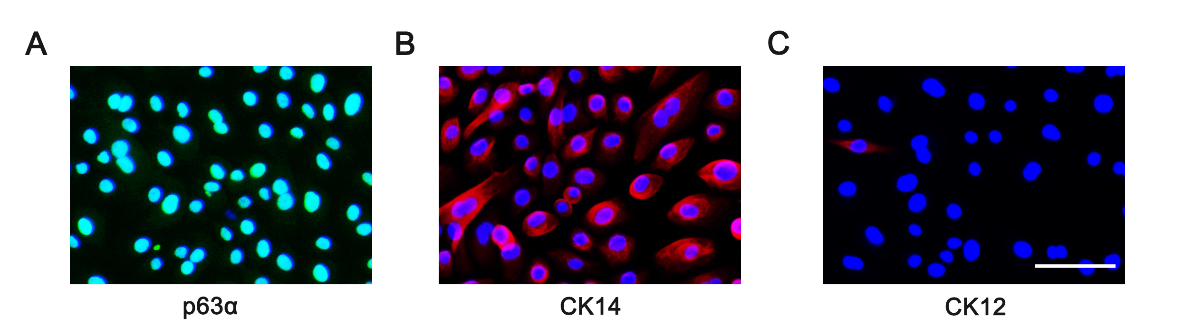


**Figure S18.** The immunofluorescent staining of LESC markers (A) p63α, (B) CK14 and differentiation marker (C) CK12 after isolation to identify primary rabbit LESCs. Scale bar: 100 μm.


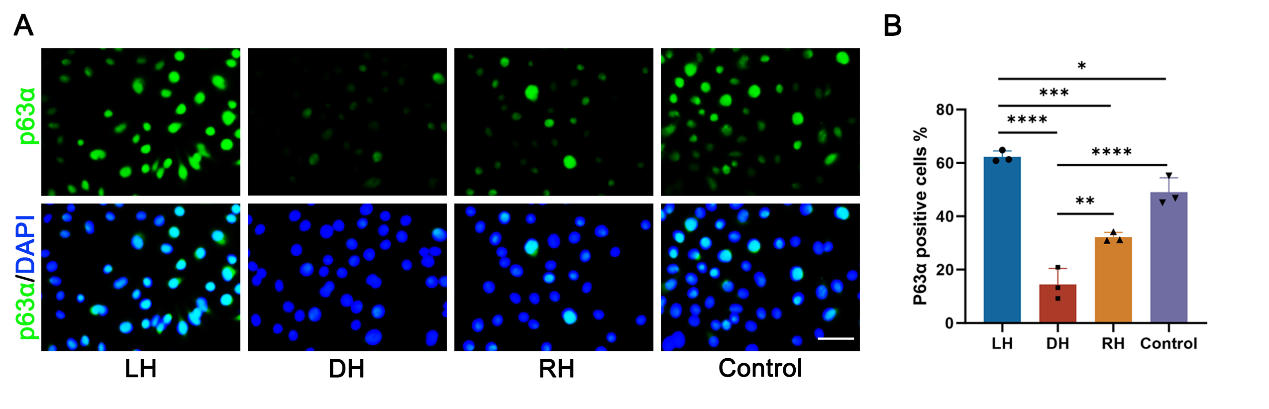


**Figure S19. The immunofluorescence staining of p63α.** (A) The representative immunofluorescence staining of p63α. (B) Statistical analysis of p63α positive cells among various groups. Scale bar: 50 μm. (n ≥ 3, * P < 0.05; ** P < 0.01; *** P < 0.001, **** P < 0.0001).


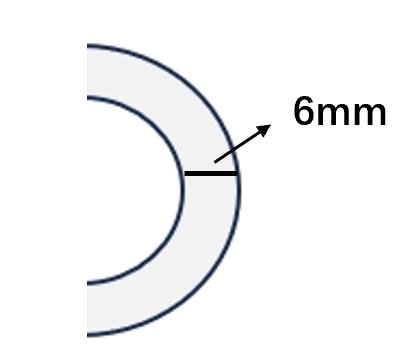


**Figure S20.** The schematic diagram of filter paper.


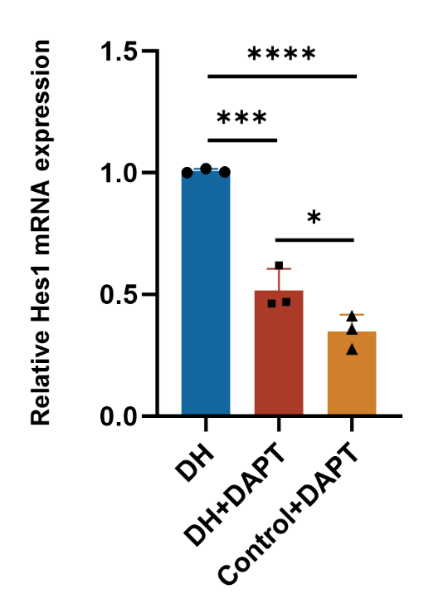


**Figure S21.** Relative Hes1 mRNA expression among various groups. (n ≥ 3, * P < 0.05, *** P < 0.001, **** P < 0.0001).

**Table S1 Primers used for quantitative polymerase chain reaction**

| Gene | Forward primers (5'->3') | Reverse primers (5'->3') |
| --- | --- | --- |
| CK3 | GCGAGGAGAGCAGGATGTC | GTGCTGCTGCTGTTGACCA |
| CK12 | GAGCTGGCCTACATGAAG | TTGCTGGACTGAAGCTGCTC |
| CK15  Notch 1  HES1  TNF-α  MMP-9  IL-1 β  IL-6  GAPDH | TGGCATCCGGGAAGTCTC  ACGAGTGTGTGAAGAACCCG  ACAGCCTCTGAGCACAGGAA  CCCTCAGGAGGAAGAGTCCC  TCCAGTACCGAGAGAAAGCC  GAGGCAAGAGGCACAACAGA  TGCACTTCAGGGTGATCGG  TCGGAGTGAACGGATTTGGC | TTCCCTGTGTCTGGCACTCA  CCGTTGTGACATGGGTTCGG  GAATGCCGCGAGCTATCTTTC  GGTTTGCTACTACGTGGGCT  GCAGTGCAGGATGTCAAAGC  CCATGGCTGTCCTAGAGATTTGA  GCCCGAGAAGCTGATCTGAG  TGCCGTGGGTGGAATCATAC |
